# Comparative genomics and pathogenicity potential of members of the *Pseudomonas syringae* species complex on *Prunus* spp

**DOI:** 10.1186/s12864-019-5555-y

**Published:** 2019-03-05

**Authors:** Michela Ruinelli, Jochen Blom, Theo H. M. Smits, Joël F. Pothier

**Affiliations:** 1 Environmental Genomics and Systems Biology Research Group, Institute for Natural Resources Sciences, Zurich University of Applied Sciences, CH-8820 Wädenswil, Switzerland; 2 Bioinformatics and Systems Biology, Justus-Liebig-University Giessen, 35392 Giessen, Germany

**Keywords:** Bacterial canker, In vitro pathogenicity test, Type III effectors, Phytotoxins, Phytohormons

## Abstract

**Background:**

Diseases on *Prunus* spp. have been associated with a large number of phylogenetically different pathovars and species within the *P. syringae* species complex. Despite their economic significance, there is a severe lack of genomic information of these pathogens. The high phylogenetic diversity observed within strains causing disease on *Prunus* spp. in nature, raised the question whether other strains or species within the *P. syringae* species complex were potentially pathogenic on *Prunus* spp.

**Results:**

To gain insight into the genomic potential of adaptation and virulence in *Prunus* spp., a total of twelve de novo whole genome sequences of *P. syringae* pathovars and species found in association with diseases on cherry (sweet, sour and ornamental-cherry) and peach were sequenced. Strains sequenced in this study covered three phylogroups and four clades. These strains were screened in vitro for pathogenicity on *Prunus* spp. together with additional genome sequenced strains thus covering nine out of thirteen of the currently defined *P. syringae* phylogroups. Pathogenicity tests revealed that most of the strains caused symptoms in vitro and no obvious link was found between presence of known virulence factors and the observed pathogenicity pattern based on comparative genomics. Non-pathogenic strains were displaying a two to three times higher generation time when grown in rich medium.

**Conclusion:**

In this study, the first set of complete genomes of cherry associated *P. syringae* strains as well as the draft genome of the quarantine peach pathogen *P. syringae* pv. persicae were generated. The obtained genomic data were matched with phenotypic data in order to determine factors related to pathogenicity to *Prunus* spp. Results of this study suggest that the inability to cause disease on *Prunus* spp. in vitro is not the result of host specialization but rather linked to metabolic impairments of individual strains.

**Electronic supplementary material:**

The online version of this article (10.1186/s12864-019-5555-y) contains supplementary material, which is available to authorized users.

## Background

Members of the *Pseudomonas syringae* species complex are hemibiotrophic plant pathogenic bacteria responsible for the development of diseases on a wide range of plant species [1–3] but have also been isolated from non-agricultural habitats such as rivers and snow [4, 5].

Within the *P. syringae* species complex, more than 60 pathovars (pv.) have been defined based on the host range [6–9] whereas nine different genomospecies were identified by DNA:DNA hybridization experiments [10], which were later reflected by phylogroups (PGs) based on similarity of housekeeping genes [11, 12]. To date, a total of thirteen PGs have been defined within the *P. syringae* species complex [13]. As revealed by previous studies, many strains isolated from non-agricultural environments were phylogenetically closely related to plant associated strains and were also shown to be pathogenic on plants such as kiwifruit and tomato [13–15].

The pathogenicity and virulence of strains belonging to the *P. syringae* species complex has been shown to mainly rely on the presence of a gene cluster called *hrp/hrc* (*hypersensitive reaction* and *pathogenicity* / *hypersensitive reaction and conserved*) [16, 17] which was found also in many other plant pathogenic bacteria such as *Xanthomonas* and *Erwinia* [18, 19]. This gene cluster encodes for a type III secretion system (T3SS) which delivers so-called type III effectors (T3E) into the host cell, where they act by suppressing the plant immune defences and promoting virulence by different and mostly unknown mechanisms [20–25]. In addition to the *hrp/hrc* cluster, a second cluster encoding for a T3SS orthologous to the T3SS of rhizobia was found to be present within the *P. syringae* species complex [26]. However, the role of this T3SS2 in pathogenicity in *P. syringae* is still unknown [26].

The production of phytotoxins by members of the *P. syringae* species complex has also been shown to play a relevant role in virulence [27]. Beside cytotoxic phytotoxins like syringomycin and syringopeptin [28–30], other phytotoxins like phaseolotoxin, mangotoxin and tabtoxin have been identified within the *P. syringae* group able to specifically interfere with the plant amino-acid biosynthesis pathways [31–34]. Syringolin is another phytotoxin produced by strains of *P. syringae* that acts as an irreversible proteasome inhibitor and promotes bacterial colonization in the apoplast by inhibiting stomatal closure [35]. Moreover, members of the *P. syringae* species complex are also able to produce chemical compounds such as auxin, cytokinins and coronatine able to mimic plant hormones and therefore specifically interfere with the regulation of plant immune response [36–39].

With the advent of affordable next-generation sequencing technologies, hundreds of whole-genome sequence assemblies belonging to *P. syringae* strains became available in the public databases. Based on this data, many comparative genomic studies have been performed among strains of different pathovars with the aim to define host-specificity related factors [11, 40, 41]. Despite being relevant to investigate host-pathogen co-evolution, such studies are rarely accompanied by a proper host-range determination of the analysed strains and therefore are less suitable for investigations of pathogenicity-related elements.

To date, the *Prunus* genus comprises the group of hosts associated with the major number of different and polyphyletic pathovars and species within the *P. syringae* species complex if compared to all other known *P. syringae* host plants. In fact, a total of six pathovars and two independent species distributed throughout PG1, PG2 and PG3 of the *P. syringae* species complex have been associated with diseases on *Prunus* spp. [1, 42–48]. Distantly related strains are found naturally associated with disease on the same host (or group of hosts), raising the question whether other strains or species within the *P. syringae* species complex, including strains isolated from non-agricultural habitats, are also potentially pathogenic on *Prunus* spp. Despite their economic significance, there is a lack of genomic information on members of the *P. syringae* species complex pathogenic to *Prunus* spp. that constitutes a real obstacle to an accurate taxonomical definition and generally to a better comprehension of these pathogens.

In this study, we generated twelve complete genomes of strains belonging to the main pathovars associated with diseases on cherry trees as well as two draft genomes of the peach quarantine pathogen *P. syringae* pv. persicae. Together with 39 additional genome sequenced strains isolated from other hosts and from non-agricultural environments, the strains used for genome sequencing in this study were tested in vitro for pathogenicity towards *Prunus* spp. Based on the pathogenicity tests results, a comparative genomics approach was applied in order to define the sets of known pathogenicity related factors such as T3E and phytotoxins present in pathogenic and non-pathogenic strains.

## Methods

### Bacterial strains and culture conditions

A set of twelve strains from the *P. syringae* species complex and isolated from diseased *Prunus* spp. hosts was selected for whole genome sequencing using PacBio RSII or Illumina MiSeq (Table 1). For comparative genomics purpose, the whole genome data obtained in this study were combined with the publicly available genome data of 39 additional strains from the *P. syringae* species complex including strains isolated from *Prunus* spp. (*n* = 3), other woody plants (*n* = 16), herbaceous plants (*n* = 13) as well as strains isolated from non-agricultural environments (*n* = 7) and were covering nine of the thirteen PGs defined by Berge et al. [13]. The 39 additional strains were selected based on 1) their availability for testing in the laboratory, 2) having a genome with less than 1000 contigs, and 3) a set of diverse hosts and PG were covered.

**Table 1:** List of strains used in this study

Strain^a^	Code	Origin	Source^b^	Host	GenBank Acc. Nr.^c^	Reference	PG
*P. syringae* pv. actinidiae ICMP 18884	Psa ICMP 18884	NZ, 2010	J. Vanneste	kiwifruit	CP011972.1	[98]	PG1b
*P. syringae* pv. actinidiae CRAFRU8.43	Psa CRAFRU8.43	IT, 2008	M. Scortichini	kiwifruit	AFTG01	[99]	PG1b
*P. syringae* pv. actinidiae ICMP 18886	Psa ICMP 18886	NZ, 2010	J. Vanneste	kiwifruit	AOJT01	[100]	PG1b
*P. syringae* pv. actinidiae ICMP 9617^P^	Psa ICMP 9617P	JP, 1984	M. Scortichini	kiwifruit	AFTH01	[101]	PG1b
*P. syringae* pv. actinidiae ICMP 19103	Psa ICMP 19103	JP, 1987	J. Vanneste	kiwifruit	AOJQ01	[100]	PG1b
*P. syringae* pv. actinidiae ICMP 19073	Psa CMP 19073	KR, 1998	J. Vanneste	kiwifruit	AOJR01	[100]	PG1b
*P. syringae* pv. theae ICMP 3923	Pth ICMP 3923	JP, 1970	CFBP	tea	LJRU01	N.A.	PG1b
*P. syringae* pv. actinidifoliorum ICMP 18883	Pfm ICMP 18883	NZ, 2010	J. Vanneste	kiwifruit	AOKH01	[100]	PG1b
*P. syringae* pv. morsprunorum race 2 CFBP 3800	Pmp2 CFBP 3800	UK, N.A.	CFBP	sour cherry	OLMQ01000001-OLMQ01000003; LT985190-LT985191^PB^	This work	PG1b
*P. amygdali* pv. morsprunorum race 2 M302280^P^	Pmp2 MAFF 302280	US, N.A.	J. Dangl	plum	AEAE01	[40]	PG1b
*P. syringae* pv. morsprunorum race 2 CFBP 6411	Pmp2 CFBP 6411	UK, 1995	CFBP	sweet cherry	LT963408^PB^	This work	PG1b
*P. avellanae* BPIC 631^T^	Pav BPCI631	GR, 1976	C. Morris	hazelnut	AKBS01	[102]	PG1b
*P. avellanae* PaVt10	Pav PaVt10	IT, 2010	C. Morris	hazelnut	JYHC01	[103]	PG1b
*P. syringae* USA007	USA007	US, 2007	C. Morris	stream water	AVDY02	[104]	PG1b
*P. syringae* CC1559	CC1559	FR, 2006	C. Morris	snow	AVEG02	[104]	PG1b
*P. syringae* pv. viburni ICMP 3963^P^	Pvi ICMP 3963	US, N.A.	CFBP	*Viburnum* sp.	LJRR01	N.A.	PG1b
*P. amygdali* pv. lachrymans M302278	Pla M302278	US, 1935	J. Dangl	cucumber	AEAM01	[40]	PG1a
*P. syringae* pv. maculicola CFBP 1657^P^	Pma CFBP1657	NZ, 1965	CFBP	cauliflower	JYHH01	[103]	PG1a
*P. syringae* pv. tomato DC3000	Pto DC3000	UK, 1960	J. Stavrinides	tomato	AE016853.1	[105]	PG1a
*P. syringae* CC1630	CC1630	US, 2007	C. Morris	onobrychis	AVED02	[104]	PG1a
*P. syringae* pv. tomato NCPPB 1108	Pto NCPPB 1108	UK, 1960	NCPPB	tomato	ADGA01	N.A.	PG1a
*P. syringae* pv. avii CFBP 3846^P^	Pavii CFBP 3846	FR, 1991	CFBP	sweet cherry	LT963402-LT963407^PB^	This work	PG1a
*P. syringae* pv. persicae CFBP 1573^P^	Ppe CFBP 1573	FR, 1974	CFBP	peach	ODAL01000001-ODAL01000214^MS^	This work	PG1a
*P. syringae* pv. persicae NCPPB 2254	Ppe NCPPB 2254	FR, 1969	NCPPB	peach	ODAM01000001-ODAM01000246 ^MS^	This work	PG1a
*P. syringae* pv. syringae CFBP 2118	Psy CFBP 2118	FR, 1979	CFBP	sour cherry	LT962481^PB^	This work	PG2d
*P. syringae* pv. syringae B728a	Psy B728a	US, 1987	J. Stavrinides	bean	CP000075.1	[106]	PG2d
*P. syringae* pv. syringae CFBP 4215	Psy CFBP 4215	FR. 1997	CFBP	sweet cherry	LT962480^PB^	This work	PG2d
*P. syringae* CC94	CC94	FR, 1997	C. Morris	cantaloupe	AVEA02	[104]	PG2d
*P. syringae* USA011	USA011	US, 2007	C. Morris	stream water	AVDX02	[104]	PG2d
*P. syringae* CC1543	CC1543	FR, 2006	C. Morris	lake water	AVEJ02	[104]	PG2b
*P. syringae* pv. avellanae ISPAVE013	Psav ISPAVE013	IT, 1992	D. Guttman	hazelnut	AKCJ01	[102]	PG2b
*P. syringae* BRIP39023	BRIP39023	AU, 1971	R. Shivas	barley	AMZX01	[107]	PG2a
*P. syringae* pv. papulans ICMP 4048^P^	Ppp ICMP 4048	CN, 1973	CFBP	apple	LJRB01	N.A.	PG2a
*P. cerasi* PL963	P.cerasi PL963	PL, 2007	M. Kałużna	sweet cherry	LT222313-LT222319 ^PB^	This work	PG2a
*P. cerasi* PL58^T^	P.cerasi PL58	PL, 2007	M. Kałużna	sour cherry	LT222313-LT222319^PB^	[46]	PG2a
*P. syringae* pv. tagetis ICMP 4091^P^	Ptg ICMP4091	ZW, 1972	CFBP	mexican marigold	LJRM01	N.A.	PG6
*P. syringae* pv. morsprunorum race 1 CFBP 3840	Pmp1 CFBP 3840	FR, 1996	CFBP	sweet cherry	LT963409-LT963413^PB^	This work	PG3
*P. syringae* pv. morsprunorum race 1 CFBP 2116	Pmp1 CFBP 2116	FR, 1974	CFBP	sour cherry	LT985192-LT985195; OLMD01000001-OLMD01000002^PB^	This work	PG3
*P. amygdali* pv. dendropanacis CFBP 3226^P^	Pde CFBP 3226	JP, 1979	CFBP	dendropanax trifidus	JYHG01	[103]	PG3
*P. amygdali* CFBP 3205^T^	P.amygdali CFBP 3205	GR, 1967	CFBP	almond	JYHB01	[103]	PG3
*P. savastanoi* pv. savastanoi DAPP-PG722	Psv DAPP-PG722	IT, 2007	C. Moretti	olive	JOJV01	[108]	PG3
*P. syringae* pv. cerasicola CFBP 6110	Pscer CFBP 6110	JP, 1995	CFBP	ornamental-cherry	OLMP01000001-OLMP01000002; LT985210-LT985212^PB^	This work	PG3
*P. syringae* pv. cerasicola CFBP 6109^P^	Pscer CFBP 6109	JP, 1995	CFBP	ornamental-cherry	LT963391-LT963394^PB^	This work	PG3
*P. amygdali* pv. aesculi 0893_23	Pae 0893_23	IN, 1969	J. Dangl	horse chestnut	AEAD01	[40]	PG3
*P. syringae* pv. phaseolicola 1448a	Pph 1448a	ET, 1985	CFBP	bean	CP000058.1	[109]	PG3
*P. syringae* CC1557	CC1557	FR, 2006	C. Morris	snow	CP007014.1	N.A.	PG10b
P. *syringae* CC1583	CC1583	FR, 2006	C. Morris	epilithon	AVEF02	[104]	PG10a
*P. cannabina* pv. alisalensis ES4326	Pal ES4326	US, 1965	J. Dangl	radish	AEAK01	[40]	PG5
*P. syringae* CC1513	CC1513	FR, 2006	C. Morris	Hutchinsia alpina	AVEL02	[104]	PG4
*P. syringae* CC1629	CC1629	US, 2007	C. Morris	oats	AVEE02	[104]	PG4
*P. syringae* CC1524	CC1524	FR, 2006	C. Morris	stream water	AVEK02	[104]	PG9
*P. viridiflava* CFBP 1590	Pvir CFBP 1590	FR, 1974	CFBP	sour cherry	LT855380	[78]	PG7

*N.A*. not available

^a^Superscript following strain names indicate ^T^ the type strain of a species and ^P^ the pathotype strain for a pathovar. Culture collections providing strains are abbreviated in the strain names as ATCC (American Type Culture Collection, Manassas, Virginia, USA), CFBP (Collection Française de Bactéries associées aux Plantes, FR), DSM (German Collection of Microorganisms and Cell Cultures, DE), ICMP (International Collection of Microorganisms from Plants, NZ), LMG (Bacteria collection of the Laboratory for Microbiology of the Faculty of Sciences of the Ghent University, BE), NCPPB (National Collection of Plant Pathogenic Bacteria, UK) and MAFF (NIAS Genebank of the Ministry of Agriculture, Forestry and Fisheries, JP)

^b^Strain obtained from Dr. Jeff Dangl (The University of North Carolina, Chapel Hill, US), Prof. Dr. David Guttman (University of Toronto, CA), Dr. Monika Kałużna and Dr. Joanna Puławska (Research Institute of Horticulture, Skierniewice, PL), Dr. Cindy E. Morris (Institut National de la Recherche Agronomique, Montfavet, FR), Dr. Marco Scortichini (Research Centre for Fruit Trees, Rome, IT), Dr. Roger G. Shivas and Yu Pei Tan (Department of Agriculture, Fisheries, and Forestry, Brisbane, AU), Dr. John Stavrinides (University of Regina, Saskatchewan, CA), Dr. Joel Vanneste (Plant and Food Research, Hamilton, NZ), CFBP (Collection Française de Bactéries associées aux Plantes, FR) or NCPPB (National Collection of Plant Pathogenic Bacteria, UK)

^c^For Whole Genome Sequence (WGS) accession numbers are provided as four letters prefixes and two digits for the version number of the data set. Superscript ^PB^ and ^MS^ following accession numbers indicate strains sequenced in this study using PacBio or MiSeq, respectively

All *P. syringae* strains used in this study were routinely grown at 28 °C on lysogenic broth (LB) agar or in LB liquid medium while shaking at 220 rpm. Most of the strains were received from collaborators as stabs or on plates. A total of 21 strains was obtained as freeze-dried samples from culture collections such as CFBP or NCPPB (Table 1) and revived according to the protocol suggested by the culture collection. The identity of strains was confirmed by *cts* amplification and Sanger-sequencing using the forward primer cts Fp 5′-AGTTGATCATCGAGGGCGCWGCC-3′ and the reverse primer cts Rp 5′-TGATCGGTTTGATCTCGCACGG-3′ published by Sarkar and Guttman [49]. Sequencing was performed at Microsynth AG (Balgach, Switzerland).

### Whole-genome sequencing and assembly

Genomic DNA for PacBio whole genome sequencing was extracted from the selected strains following the protocol described elsewhere [50]. PacBio library preparation and sequencing were performed at the Functional Genomic Center Zurich. SMRTbells were prepared using the DNA Template Prep Kit 2.0 (3 kb to 10 kb) (Pacific Biosciences, Menlo Park, CA) and sequencing was performed on a PacBio RSII system (Pacific Biosciences) run with a P4/C2 chemistry using five to six SMRTcells per strain. Reads were assembled on the SMRT analysis software platform version 2.3.0 using the Hierarchical Genome Assembly Process (HGAP3) protocol followed by manual assembly using BLAST or the SeqMan Pro subroutine of the Lasergene Package (DNASTAR, Madison, WI). Genomic DNA for whole genome shotgun sequencing using Illumina MiSeq (Illumina, San Diego, CA) was extracted with the NucleoSpin Tissue Kit (Macherey-Nagel AG, Düren, DE) following the manufacturer’s protocol. The library preparation was then performed on an Illumina NeoPrep System (Illumina) with a TruSeq Nano DNA kit (Illumina) according to manufacturer’s instructions with six PCR cycles. Paired-end sequencing of 300 bp was performed using MiSeq Reagent Kit v.3 (Illumina) following manufacturer’s instructions. Automatic assemblies were performed using SPAdes Genome Assembler v.3.5.0 [51] on a BaseSpace Onsite v.2.1.2 (Illumina). Putative plasmids were identified by the presence of self-closing molecules during assemblies and/or of genes involved in plasmid replication or mobilization.

### Phylogenomics

Automatic genome annotation of the sequenced strains was performed using the GenDB platform v.2.4 [52]. The core genome phylogenetic relationships were obtained using EDGAR v.2.2 [53]. Briefly, the core genome was defined by iterative pairwise comparison of the gene content of each of the selected genomes using the bidirectional best hits (BBH) as orthology criterion. For all calculations, protein BLAST (BLASTp) was used with BLOSUM62 as similarity matrix [54, 55]. Genes were considered orthologous when a reciprocal best BLAST hit was found between two genes, and when both BLAST hits were based on alignments exceeding 70% sequence identity spanning over at least 70% of the query gene length [56]. Multiple alignments of each orthologous gene set of the core genome were calculated using the MUSCLE software [57] and non-matching parts of the alignments were removed based on GBLOCKS [58]. The resulting alignments were concatenated and used to construct a Neighbour Joining (NJ) phylogeny as implemented in the PHYLIP package [59]. Non-annotated genomes retrieved from the NCBI database were annotated using a command line annotation pipeline based on HMMer against an EDGAR based database of *Pseudomonas* ortholog groups followed by reference genome annotation and a comparison to the Swiss-Prot and RefSeq databases for genes that had no high quality hit in previous steps [60]. In addition to the core-genome phylogeny, the average nucleotide identity based on BLASTn (ANIb) values were calculated between each genome using EDGAR v2.2 [60].

### Pathogenicity tests on immature cherry fruitlets

Pathogenicity tests on immature cherry fruitlets were performed following the protocol described elsewhere [61]. Freshly collected immature sweet cherry fruitlets (cv. Christiana × Gisela5) were dipped in 50% ethanol for 3 min and rinsed three times with sterile distilled water. All tested strains (*n* = 51, Table 1) were grown overnight in liquid LB medium at 28 °C while shaking at 220 rpm. Bacteria were collected by centrifugation and washed twice with sterile distilled water. Final bacterial concentration was adjusted to OD_600_ = 0.5 (corresponding to around 10^8^ CFU/ml) with sterile distilled water. For each strain, ten fruitlets were inoculated by pricking in two places on the fruitlet with a sterile needle previously immersed in the bacterial suspension. Sterile distilled water was used as negative control. After inoculation, the fruitlets were put on a moist sterile filter paper into a Petri dish, sealed with parafilm and incubated at 22 °C for four days in the dark. Pathogenicity was assessed visually looking at the symptoms developed at the pricking sites.

### Detached leaf bioassay

The detached leaf bioassay was performed as described elsewhere [62] with some slight modifications. Leaves from *Prunus persica* cv. Red Haven and from *Prunus dulcis* cv. Dürkheimer Riesenmandel were freshly collected and washed for 5 min under running tap water, dipped into 70% ethanol for 1 min and then into a 6.5% sodium hypochlorite solution for 5 min. After disinfection, leaves were rinsed three times in sterile distilled water and air-dried under a sterile flow bench. All tested strains (*n* = 24) were grown overnight in liquid LB medium at 28 °C while shaking at 220 rpm. Bacteria were collected by centrifugation and washed twice with sterile 0.8% KCl. Final concentration was adjusted to OD_600_ = 0.5 with sterile 0.8% KCl.

Leaves were infiltrated from the abaxial leaf side with the bacterial suspension using a sterile disposable 3 ml syringe without needle applying a gentle pressure until the mesophyll tissue became water soaked. Each leaf was infiltrated with eight to ten different strains including the positive and the negative control (i.e. *P. syringae* pv. syringae strain CFBP 2118 and 0.8% KCl, respectively). Every strain was infiltrated once into three different leaves. Each inoculated leaf was placed into a Petri dish containing water agar (10 g/L) sealed with parafilm and incubated for one week at 25 °C under daylight photoperiod. A strain causing the formation of a clear brownish necrotic spot on the site of infiltration for all three infiltrated leaves was considered as pathogenic.

### Bacterial growth assays

All growth curves were obtained using the Bioscreen C Microbiology Analyser (Oy Growth Curves AB Ltd., Helsinki, Finland). For this purpose, bacteria were grown overnight in liquid LB medium at 28 °C while shaking at 220 rpm. Bacterial cells were then collected by centrifugation (10 min at 3220 x *g*), washed three times with sterile 0.8% KCl and finally diluted to an OD_600_ = 0.01 with LB. Each strain was tested in triplicates.

### Comparative genomics of known virulence related factors

In order to determine the virulence factors profile of the selected strains, the locus tags of the corresponding amino acid sequences were obtained from the NCBI database (Additional file 1: Table S1) and used as query to screen the remaining genomes for orthologous proteins using EDGAR v2.2 [53]. For the T3E screening, the amino acid sequence of a total of 80 T3E was obtained from the Hop database available at the *Pseudomonas syringae* Genome Resources website (www.pseudomonas-syringae.org) and used as query in a tBLASTn analysis to retrieve the corresponding locus tags to be used in EDGAR v2.2 [53] to search for the reciprocal best hit on the selected genomes (*n* = 51) (Additional file 1: Table S2).

## Results

### Genome sequencing and assembly

De novo assembly of PacBio reads yielded a total of contigs ranging from one to seven with a mean coverage of over 100× for each of the genomes (Additional file 1: Table S3). The size of the chromosome ranged from 5.8 Mb to 6.4 Mb and with an average G + C content of 58.6% ± 0.5% for the sequenced chromosomes whereas putative plasmids ranged from 20 kb to 140 kb and generally displayed a lower G + C content (~ 55%) (Additional file 1: Table S3). Automatic genome annotation predicted a total number of coding sequences (CDS) varying between 5118 and 5995 (Additional file 1: Table S3). The whole genome sequencing of the *P. syringae* pv. persicae strain CFBP 1573 using Illumina MiSeq yielded a total of 214 contigs and a mean coverage of 61× (Additional file 1: Table S4). Similar results were obtained for the *P. syringae* pv. persicae strain NCPPB 2254 with a total 246 contigs and mean coverage of 43×. Both genomes had a total size of 6.4 Mb and a G + C content of 58% (Additional file 1: Table S4). The number of CDS predicted using GenDB was 6079 and 5990 for strains CFBP 1573 and NCPPB 2254, respectively.

### Phylogenomics

**Fig. 1:**
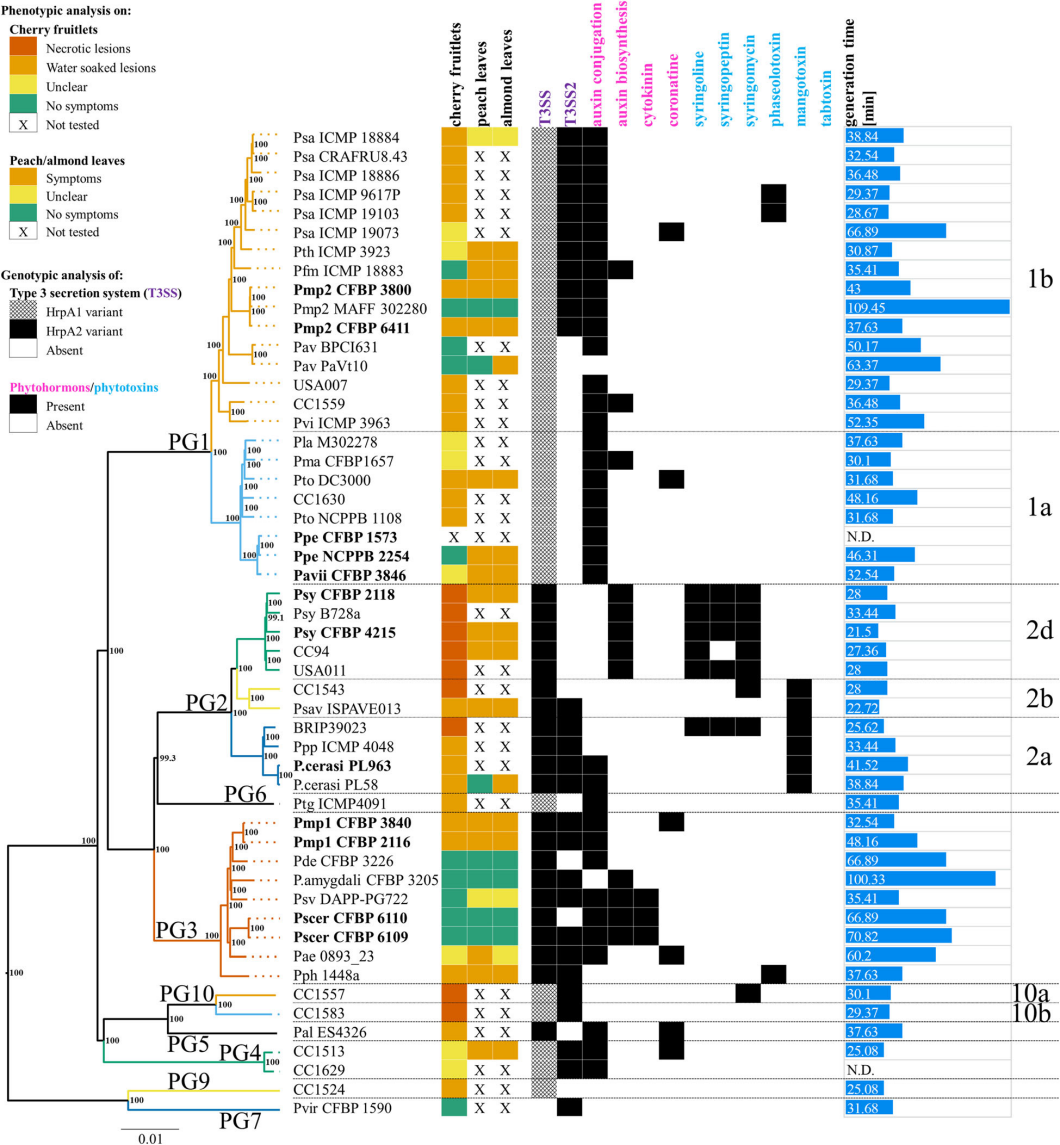
Pathogenicity tests results and virulence factors profile of the analyzed *Pseudomonas syringae* strains. Strains sequenced in this study are indicated in bold. Strains are ordered based on the core-genome. Approximately Maximum-Likelihood phylogenetic tree constructed on the similarity of 2085 protein sequences corresponding to a total alignment length of 840,202 amino acids per genome. The local support values computed using the Shimodaira-Hasegawa test are indicated close to the nodes. The tree was generated using EDGAR v.2.2 [53]. The strain names refer to the code field from Table 1. Phylogroups (PGs) are reported on the left and are separated by horizontal dashed lines whereas clades are reported on the right and are separated by horizontal dotted lines. Results of the pathogenicity tests performed on immature cherry fruitlets, peach and almond detached leaves are reported in the first three columns (see graphical legend). No pathogenicity test was performed for strains displaying a X sign in a white square. A strain was defined as possessing T3SS2, a second cluster encoding for a type III 3 secretion system (T3SS) homologous to the one found in rhizobia, if at least 22 out of the 27 genes constituting this system were retrieved. Presence (black) and absence (white) of clusters for biosynthesis and regulations of the known phytohormones (pink) and phytotoxins (blue) is also reported. The generation time in hours was derived from the slope of the logarithmic (log_10_) growth curve. IaaM (tryptophan monooxygenase) and IaaH (indoleacetamide hydrolase) are responsible for the synthesis of auxin whereas IaaL (indole-acetic acid-lysine synthase) is conjugating auxin to lysine decreasing the concentration of the active form of auxin. Locus tags used for the genotypic screening are reported in Additional file 1: Tables S1 and S2. N.D.: not determined

In order to clarify the exact phylogenetic position of the sequenced *Prunus* associated strains within the *P. syringae* species complex a core genome based phylogeny was generated using EDGAR v.2.2 [53]. The obtained tree was generated based on the concatenated and aligned amino acid sequences of 2085 proteins consisting of a total length of 840,202 amino acids (Fig. 1). The main clustering obtained from the core genome phylogeny reflected the PGs previously defined by Multi Locus Sequence Analysis (MLSA) [11, 49, 63] and single locus phylogeny [12, 13]. The sequenced *Prunus* associated strains fell into three different PGs namely PG1 (*P. syringae* pv. morsprunorum race 2, *P. syringae* pv. avii, *P. syringae* pv. persicae), PG2 (*P. syringae* pv. syringae and *P. cerasi*) and PG3 (*P. syringae* pv. morsprunorum race 1 and *P. syringae* pv. cerasicola). However, strains of different *Prunus* associated pathovars from the same PG did not form a monophyletic group (Fig. 1). Within PG1, *Prunus* associated strains were found in two separate clades: one with strains of the *P. syringae* pv. morsprunorum race 2 (PG1b) and one with *P. syringae* pv. persicae and *P. syringae* pv. avii (PG1a). Strains of *P. syringae* pv. syringae and *P. cerasi* were both belonging to the PG2 but clustered within PG2d and PG2a, respectively (Fig. 1). Sequenced strains of the same pathovar mostly tightly clustered with exception of the two *P. syringae* pv. syringae strains CFBP 2118 and CFBP 4215 which clustered closer to strains isolated from other hosts than to each other. The core-genome phylogeny was supported by ANIb results which revealed additionally that PGs boundaries within the *P. syringae* species complex, with ANIb values < 95%, actually represent species boundaries [64] (Additional file 1: Figure S1).

### In vitro pathogenicity tests of members of the *P. syringae* species complex on *Prunus* spp.

**Fig. 2:**
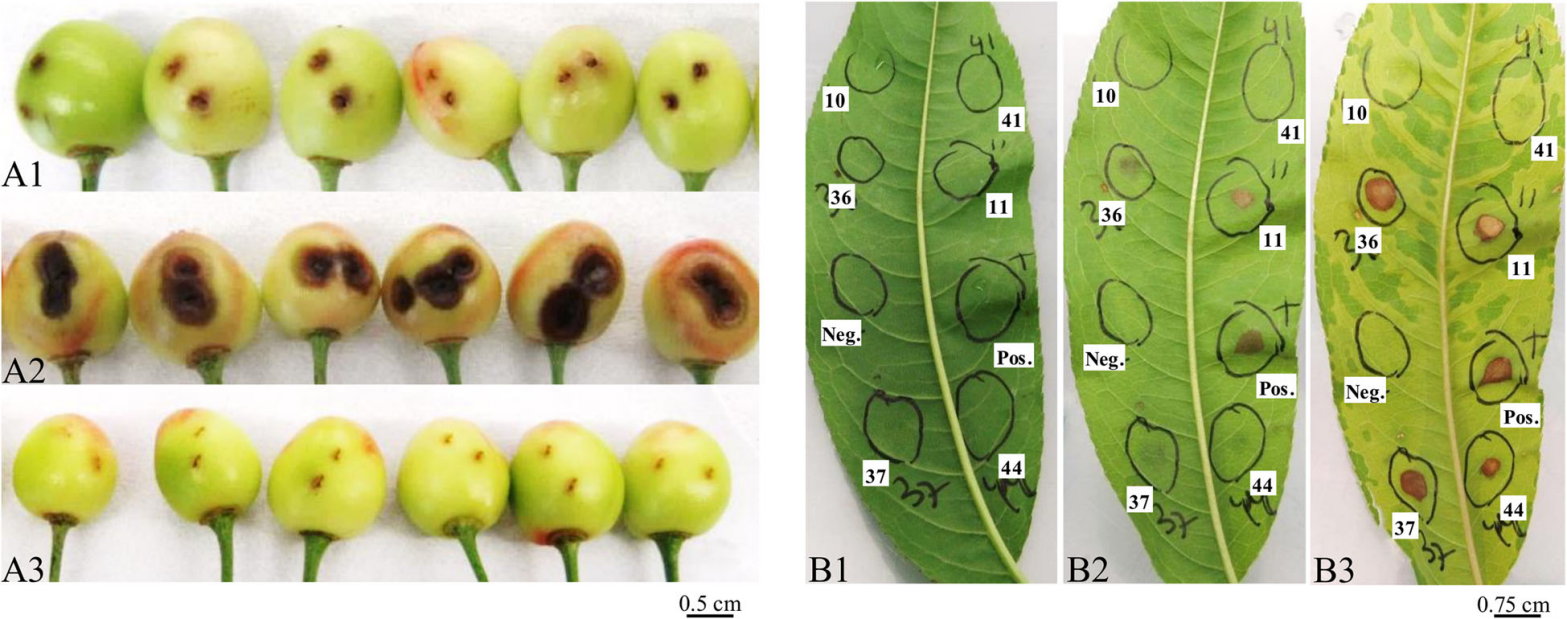
Representative results of the pathogenicity tests on cherry immature fruitlets (A) and on peach detached leaf (B). (A) Typical symptoms observed two days post inoculation with suspensions of (A1) *Pseudomonas syringae* pv. morsprunorum race 2 CFBP 2116, (A2) *P. syringae* pv. syringae CFBP 2118 and (A3) with sterile distilled water. (B) Typical results obtained at (B1) 0 days post inoculation (dpi), (B2) 2 dpi and (B3) 7 dpi during the detached leaves assays performed on peach (*Prunus persica* cv. Red Haven) infiltrated with suspensions of “10”: *P. syringae* pv. morsprunorum race 2 M302280; “41”: *P. syringae* pv. cerasicola CFBP 6110; “36”: *P. syringae* pv. morsprunorum race 1 CFBP 3840; “11”: *P. syringae* pv. morsprunorum race 1 CFBP 6411; “37”: *P. syringae* pv. morsprunorum race 1 CFBP 2116; “44”: *P. syringae* pv. phaseolicola 1448a; “Pos.”: *P. syringae* pv. syringae CFBP 2118 and “Neg.”: 0.8% KCl

Results of the cherry immature fruitlets revealed a high pathogenicity potential towards *Prunus* spp. within the *P. syringae* species complex. In particular, of the 51 strains tested on immature cherry fruitlets, 32 strains were pathogenic, ten were classified as non-pathogenic whereas nine strains were associated with unclear phenotypes, i.e. neither necrotic nor water soaked (Fig. 1). Most of the pathogenic strains (*n* = 23) caused brownish, water-soaked superficial lesions similar to those caused by *P. syringae* pv. morsprunorum race 1 and race 2 whereas the remaining strains (*n* = 9) caused formation of black-brown sunken necrotic lesions which are typically caused by *P. syringae* pv. syringae (Fig. 2A). Most of the non-pathogenic strains belonged to PG1 (*n* = 5) and PG3 (*n* = 4) and included six strains originally isolated from *Prunus* spp. like the peach pathogen *P. syringae* pv. persicae NCPPB 2254 and the *P. syringae* pv. morsprunorum race 2 pathotype strain M302280 (Fig. 1). Results obtained from the detached leaf assays (Fig. 2B) on peach and almond were mostly congruent with the virulence profile obtained from the cherry immature fruitlets inoculation but additionally revealed few strains possessing a narrower host range. In fact, *P. syringae* pv. persicae NCPPB 2254 and *P. syringae* pv. actinidifoliorum ICMP 18883 were both non-pathogenic on cherry fruitlets but were clearly pathogenic if inoculated on peach and almond leaves. Additionally, *P. cerasi* PL58 was non-pathogenic on peach leaves but showed symptoms on cherry immature fruitlets and almond leaves as well (Fig. 1). Furthermore, the *P. avellanae* strain PaVt10 was not pathogenic on cherry fruitlets and peach leaves but was symptomatic on almond leaves. Only five strains resulted to be non-pathogenic in all three in vitro pathogenicity tests, namely *P. syringae* pv. morsprunorum race 2 strain M302280 (PG1), *P. amygdali* pv. dendropanacis CFBP 3226 (PG3), *P. amygdali* CFBP 3205 (PG3) and both *P. syringae* pv. cerasicola strains CFBP 6109 and CFBP 6110 (PG3).

### Distribution of known virulence-related factors

In order to investigate a potential link between known virulence-related factors and the observed pattern of pathogenicity, the publicly available genomes of the strains selected for this study (Table 1) were screened for the presence of clusters of genes known to be involved in pathogenicity of *P. syringae*.

All strains possessed a complete *hrp/hrc* cluster (Fig. 1), with exception of *P. viridiflava* CFBP 1590, which lacked most of the genes within this cluster. The distribution of the T3SS2 among the strains considered in this study was not consistent with the PG defined based on core-genome phylogeny and even varied among strains of the same pathovar (Fig. 1). Moreover, the presence of the T3SS2 could not explain the pathogenicity profiles obtained in this study (Fig. 1).

**Fig. 3:**
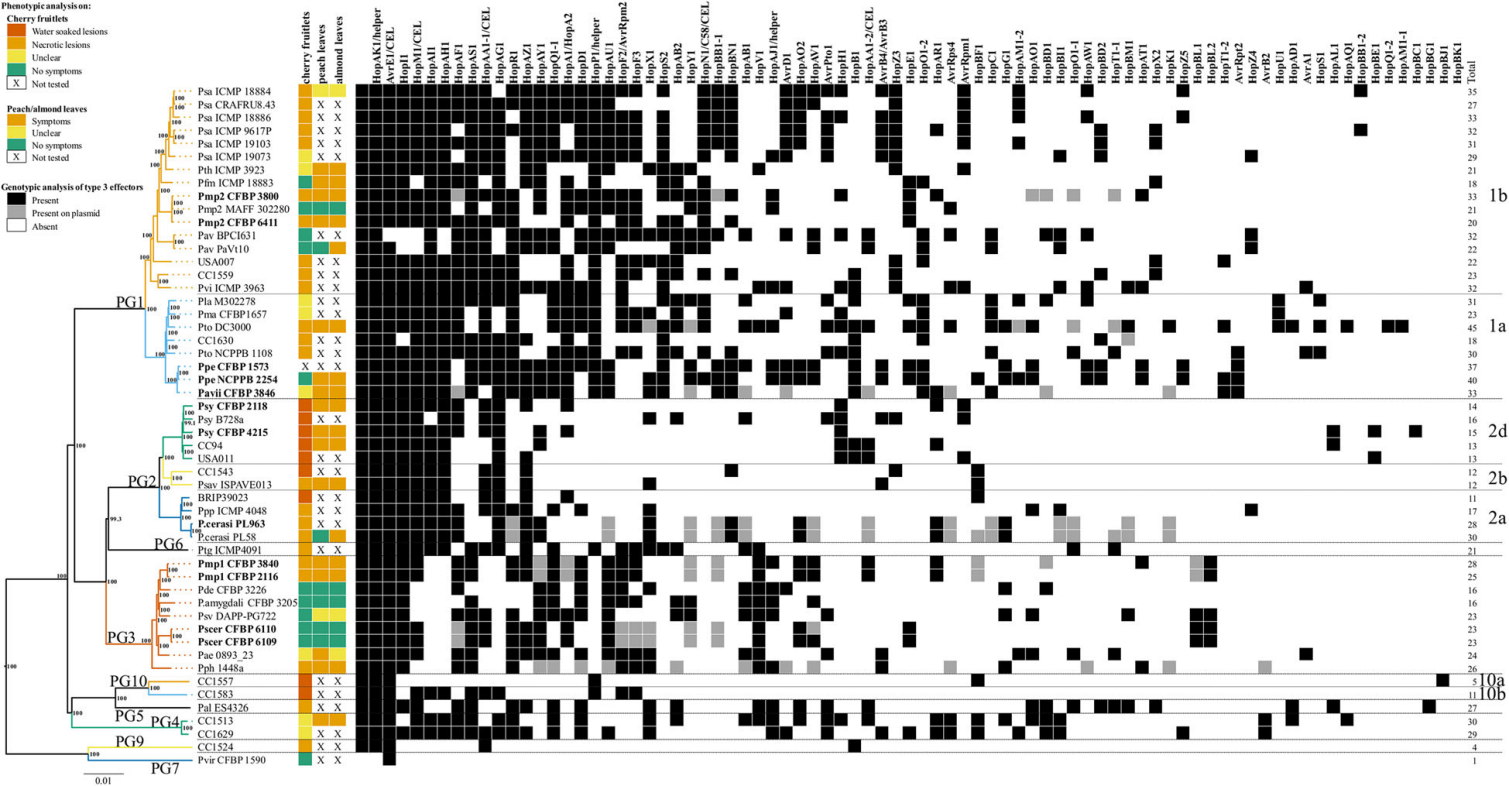
Type III effector (T3E) profile of the 52 *Pseudomonas syringae* strains used in this study. Strains sequenced in this study are indicated in bold. The amino acid sequence of a total of 80 T3E (Additional file 1: Table S2) was obtained from the Hop database available at the *P. syringae* Genome Resources website (www.pseudomonas-syringae.org) and used as query in a tBLASTn analysis to retrieve the corresponding locus tags to be used in EDGAR v.2.2 [53] for search of the reciprocal best hit on the selected genomes. Black squares indicate presence whereas white squares indicate absence of the T3E. For strains sequenced in this study using PacBio RSII as well as for the complete genomes *P. syringae* pv. tomato DC3000 and *P. syringae* pv. phaseolicola 1448a, grey squared indicates T3E located on plasmids. Strains are ordered based on the core-genome phylogeny constructed in Fig. 1 together with phenotypical analysis whereas T3E are ordered based on their abundance from left to right in descending order. The strain names refer to the code field from Table 1. Phylogroups are reported on the left and are separated by horizontal dashed lines whereas clades are reported on the right and are separated by horizontal dotted lines. The last column indicates the total number of T3E per strain. CEL: the T3E located in the conserved effector locus

Using in silico screening for 80 known T3E (Additional file 1: Table S2), the total number of T3E retrieved per strain range from one in the T3SS-impaired *P. viridiflava* CFBP 1590 to 45 found in the genome of the *P. syringae* pv. tomato DC3000. The overall T3E presence/absence distribution profile mostly reflected the core-genome phylogeny: closely related strains possessed similar T3E repertoires with some rearrangements. Also here, the T3E profiles could not explain the pathogenicity results. It was noticed that the T3E HopAA, which is located in the conserved effector locus (CEL) was absent in the genomes of the five strains that were non-pathogenic on cherry, peach and almond. However, HopAA was also missing in the genomes of *P. syringae* pv. phaseolicola 1448a and in *P. syringae* pv. aesculi 0893_23, which, in contrast, were pathogenic. As already noticed by Lindeberg et al. [65], the number of T3E present in strains from the PG2 is generally lower in comparison to strains of PG1 or PG3 (Fig. 3). However, both *P. cerasi* strains, belonging to PG2a, possessed almost the double number of T3E when compared to all other members of the PG2 while most of the T3E in *P. cerasi* were located on plasmids (Fig. 3). On the other hand, the presence of clusters for the synthesis of the necrosis-inducing phytotoxins syringomycin and syringopeptin co-occurred with the phenotype obtained from the immature cherry fruitlets assay: with exception of *P. syringae* CC1583 (PG10b), the strains causing necrotic lesions (Fig. 1) possessed clusters related to the production and regulation of syringomycin, syringopeptin or both. These clusters were generally found in strains of PG2, which were also shown to possess a lower amount of T3E (~ 20 T3E per strain). However, *P. syringae* strain CC1557, belonging to the quite distantly related PG10a, also possessed the syringomycin cluster. The syringolin cluster was exclusively found in strains from the PG2 and mostly within the PG2d clade whereas the phaseolotoxin cluster was only present in *P. syringae* pv. phaseolicola 1448a (PG3) and two strains of *P. syringae* pv. actinidiae (PG1). The mangotoxin cluster was restricted to strains from the PG2 and specifically found within the clades PG2a and PG2b (Fig. 1). The genes involved in the synthesis of the plant hormone auxin (indoleacetic acid, IAA), *iaaM* and *iaaH*, encoding respectively the tryptophane monooxygenase and IAA hydrolase were found in strains belonging to PG2d and PG3, but as well as in some strains within PG1 (*n* = 3). In contrast, the *iaaL* gene encoding the IAA-lysine synthase responsible for the reversible inactivation of IAA was found throughout the entire phylogenetic tree. The cluster for the biosynthesis of coronatine was found only in six distantly related strains whereas only three closely related strains within PG3 were potentially able to produce cytokinins.

Again, the presence of known pathogenicity factors was not related to the differences in virulence on cherry, peach and almond. Indeed, most of the analysed genes or gene clusters mainly reflected the core-genome phylogeny and could not reveal why closely related strains differed in their pathogenicity towards the tested hosts.

### Divergence of the HrpA protein among the *P. syringae* species complex

**Fig. 4:**
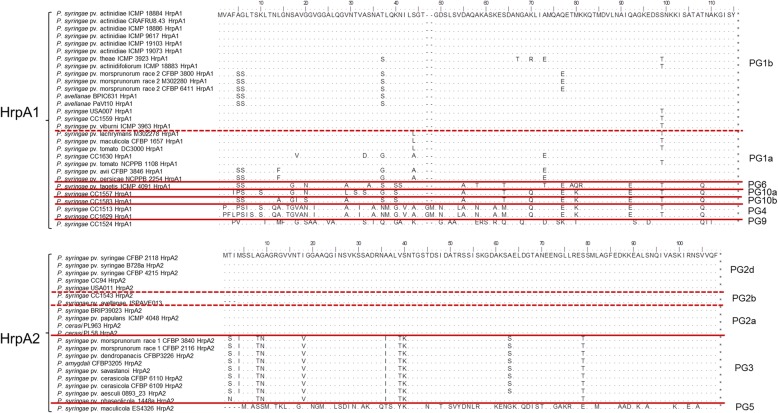
Alignment of the HrpA1 and HrpA2 proteins retrieved from the 51 genomes of members of the *Pseudomonas syringae* species complex analyses in this study. Red lines indicate the phylogroup (PG) borders, whereas red dashed lines indicate clade borders. Amino acids are only reported if different from the reference sequences (GenBank Accession no. AKT31917 and CFBP2118_03968, respectively) which are entirely displayed in the top line of each alignment

The *hrpA* gene within the *hrp/hrc* cluster encodes for the extracellular pilus of the T3SS, which is essential for a functional T3SS and has been shown to be under diversifying selection [66]. Two homologous HrpA proteins were found within the *P. syringae* species complex: one variant was found in strains of PG1, PG6, PG9 and PG10 and named HrpA1 (for HrpA like PG1) whereas the other variant was present in strains belonging to PG2, PG3 and PG5 and named HrpA2 (for HrpA like PG2) (Fig. 4). The pattern of distribution of these two HrpA variants did not reflect the core genome phylogeny. In fact, the genome of PG6 strain *P. syringae* pv. tagetis ICMP 4091 contained the HrpA1 variant gene, but the strain was phylogenetically positioned equidistantly to PG2 and PG3 strains, which both have the HrpA2 variant. The same situation was observed for the PG5 strain *P. cannabina* pv. alisalensis ES4326 (Fig. 1). The sequence analysis of HrpA1 revealed a higher level of polymorphism within strains of the same PG if compared to HrpA2 (Fig. 4). Polymorphisms of HrpA1 and HrpA2 mostly reflected the PGs but HrpA1 within strains of the PG1 displayed some more specific polymorphisms, which were generally shared among strains of the same pathovar. However, the HrpA1 protein from *P. syringae* pv. tomato NCPPB 1108 (PG1a) was identical to the HrpA1 protein of strains belonging to PG1b including strains isolated from water and snow (Fig. 4). Strains of the PG2 possessed a 100% identical HrpA2 protein and within PG3, the HrpA2 sequence was almost identical independent of the pathovar (Fig. 4).

### Potential link between pathogenicity and growth rate

The results obtained from the comparative genomics of known virulence related factors did not reveal any direct link with the results obtained from the pathogenicity tests. However, it was noticed that non-pathogenic strains usually grew at a lower growth rate in rich medium (LB) if compared to their closely related pathogenic strains (Fig. 1). For example, the non-pathogenic strain *P. syringae* pv. morsprunorum race 2 M302280 displayed a generation time of 100 min which is three times higher than what was observed for the two pathogenic *P. syringae* pv. morsprunorum race 2 strains analysed in this study. The same trend was observed with the pathogenic and non-pathogenic strains of the PG3 (Fig. 1). This suggested that a metabolic impairment could be a potential reason why those strains were not pathogenic within the timeframe of the experiments. Comparative genomics between closely related pathogenic and non-pathogenic strains revealed some mutations affecting genes involved in metabolic pathways in non-pathogenic strains, which were previously shown to be related to virulence of plant pathogenic bacteria (Table 2).

**Table 2:** List of inactivated nutrient assimilation genes in the identified non-pathogenic *Pseudomonas syringae* strains

Pathway	Gene	Pmp2CFBP 3800	Pmp 2 M300280	Pde CFBP 3226	Pamygdali CFBP 3205	Pathway is relevant for pathogenicity of:	Reference
Alginate biosynthesis	*alg8*	PSCFBP3800_01492	1-bp insertion, frame shift	+	+	*P. syringae* pv. syringae	[94]
α- ketoglutaric acid uptake	*kgtP*	PSCFBP3800_04544	+	4-bp insertion, frame shift	+	*Xanthomonas oryzae* pv. oryzae	[95]
Sugar alcohol utilization	*mtlR*	PSCFBP3800_03115	+	transposase insertion	+	*Erwinia amylovora*	[96]
Malate:quinone oxidoreductase	*mqo2*	PSCFBP3800_03180	+	transposase insertion	transposase insertion	*P. syringae* pv. tomato	[97]

“+”: same as Pmp 2 CFBP 3800

## Discussion

A prerequisite for the development of effective and targeted control measures against plant diseases is the comprehension of the mechanisms adopted by the pathogen for successful host infection.

Bacterial canker caused by members of the *P. syringae* species complex on *Prunus* spp. is responsible for relevant yield losses in both fruit and wood production worldwide [67, 68]. However, with the exception of few comparative genomics studies of pathogens on *Prunus* spp. [40, 69, 70] the repertoire of pathogenicity related factors in *Prunus* spp. associated strains remains largely unstudied. Taking advantage of the complete as well as the draft genomes generated in this study and combining them with a consistent set of publicly available genomes, we generated a whole genome based phylogeny of the *P. syringae* species complex comprising all known pathovars and species that have ever been associated with diseases in *Prunus* spp. (status April 2017), including the newly described *P. cerasi* species [46] and the quarantine peach pathogen *P. syringae* pv. persicae.

The methodology used in this study to test pathogenicity relied on two different in vitro assays, i.e. the use of detached immature fruitlet and detached leaf assays, which were previously shown to be reliable for cherry [61, 62, 70] but also for other woody hosts [70, 71]. While the use of detached organs instead of the whole plant could potentially affect the results of pathogenicity tests, the pattern of pathogenicity retrieved from this study is largely congruent with the patterns obtained from the inoculation of whole plants (C.E. Morris, personal communication), therefore supporting the veracity of the results. Nevertheless, it might be necessary to repeat the pathogenicity tests to further validate the results. Furthermore, the possibility to co-inoculate different strains together with the positive and negative controls was crucial to reduce the potential effect of physiological variation of leaves. The large number of potentially pathogenic stains retrieved from this study and the fact that strains belonging to the same pathovar varied in their pathogenicity towards *Prunus* spp. highlighted the importance of a proper host range determination in order to perform reasonable comparative genomics studies, especially if intended to investigate factors involved in host-specificity. Indeed, it is important to consider that a strain never isolated from a particular host could still be pathogenic on that host, as also previously shown for the *P. syringae*-kiwifruit and *P. syringae*-tomato pathosystems [14, 15]. At the same time, these findings revealed the weakness of the pathovar designation system for *P. syringae* taxonomy. A clear example is constituted by the two races of *P. syringae* pv. morsprunorum, whose ANIb values (~ 88%) are clearly below the species boundaries of 95% [64]. This indicates that they rather should be considered as separate species. Therefore, the genomic data supports the claims to revise the taxonomic position of the *P. syringae* species complex [72].

The results from this study also revealed that strains isolated from water reservoirs such as stream water and snow could potentially constitute a threat for *Prunus* spp. plantations, supporting the direct link between the agricultural and non-agricultural habitats occupied by *P. syringae* as already reported [4, 14, 15, 73–75]. These findings also provides some important hints for cultural practices implementation especially regarding the maintenance and hygiene of water-irrigation systems. In fact, due to the persistence of potentially pathogenic *P. syringae* strains in water basins, the use of closed (i.e. recirculating) irrigation systems should be avoided and if possible the water should be disinfected or sterilized prior to use to prevent the spread of this pathogen within plantations [76].

A first systematic screen and comparison of known virulence related factors in strains associated with *Prunus* spp. was performed in this study, revealing a high variability in the set of virulence factors comprising both T3E set as well as phytotoxins and phytohormons production. This observation led to the conclusion that pathogenicity on *Prunus* spp. may be achieved by different and currently unknown mechanisms that could not be detected in this study as we only used already known virulence related factors. However, the high level of susceptibility observed for this group of hosts to members of the *P. syringae* species complex could reflect the lack of proper defense mechanisms in the host rather than the evolution of specific virulence strategies in the pathogen. In fact, the results of our comparative genomics approach did not show an obvious match with the results obtained from the pathogenicity tests even though the description of potential link to woody compounds in the past [70]. The only exception was *P. viridiflava* strain CFBP 1590 where the absence of pathogenicity can be related to the lack of a complete T3SS combined with an extremely reduced T3E repertoire [77, 78].

We confirmed that strains possessing a small T3E repertoire were potential producers of necrosis-inducing phytotoxins like syringomycin and/or syringopeptin [40, 65] and belonged mostly to PG2 [65]. In addition, strains of PG9, PG10a and PG10b were found to possess the genes necessary for the production of at least one of those phytotoxins [13]. Nevertheless, only strains of PG2 possessed both syringomycin and syringopeptin clusters, which were previously shown to be physically linked and located on a 155-kb genomic island [79, 80]. The observed mutually exclusive presence of clusters for production of necrosis-inducing phytotoxins versus the evolution of large T3E repertoires reveals a potential trend of pathogenesis subgroup specialization within the *P. syringae* species complex with strains of the PG2 adopting a more generalist pathogenicity strategy and most of the remaining PGs relying on a specific host-targeted pathogenicity mechanism. This specialization is reflected by the broad vs. narrow host range observed within the *P. syringae* species complex [81, 82]. In contrast to the T3E, phytotoxins were never recognized by the host resulting in a non-compatible interaction. Therefore, in a specific environment, a pathogenicity mechanism relying on necrosis-inducing phytotoxins would theoretically be selectively more advantageous than the production of specialized T3E, which could lead to the induction of plant immunity [83, 84]. However T3E, phytohormons and other non-necrosis inducing phytotoxins are related to a more precise modulation of the host-physiology [31, 85] and could promote the long-term survival of bacterial populations in the host-plant which can serve as source of inoculum for further infection [86, 87].

Nevertheless, syringomycin and syringopeptin were shown to be the major virulence determinant for *P. syringae* pv. syringae strain B301-D in vitro [88], highlighting the fact that for strains possessing necrosis-inducing phytotoxins, the T3SS/T3E strategy may be of secondary importance. This would also be congruent with the observation that the HrpA protein among PG2 strains is 100% identical suggesting that low diversifying selection acts on that gene in contrast to the HrpA found in PG1 strains [66]. In addition, the HrpA protein is conserved also within strains of PG3, but strains from this PG have a narrower host range when compared to *P. syringae* pv. syringae strains [45, 47, 89].

A positive trend was observed between the necrotic phenotype on cherry immature fruitlets and presence of clusters for the production of syringomycin and syringopeptin [88]. However, while the PG10b strain *P. syringae* CC1583 was lacking the syringomycin and syringopeptin clusters, it was associated with necrotic lesions in cherry fruitlet tests, suggesting that this strain is probably able to produce another necrosis inducing phytotoxin, which is still not characterized. Although strains within PG2 usually possessed a smaller set of T3E (~ 15), both *P. cerasi* strains constituted an exception having almost the double of T3E. As we obtained high quality genomes for those strains, it was possible to determine that around half of those T3E were located on plasmids (Fig. 3) thereby highlighting the importance of horizontal gene transfer in *P. syringae* [82, 90].

Strains of the quarantine peach pathogen *P. syringae* pv. persicae are known to produce a necrosis inducing phytotoxin called persicomycin [91]. However, no necrotic phenotype was observed on cherry fruitlets inoculated with *P. syringae* pv. persicae NCPPB 2254. As persicomycin production was shown to be thermoregulated [91] it is possible that it was not induced under the used assay conditions. Moreover *P. syringae* pv. persicae NCPPB 2254 was never tested previously for production of persicomycin. On the other hand, the *P. syringae* pv. persicae pathotype strain CFBP 1573 which was shown to produce persicomycin under in vitro conditions [91] did not cause necrotic lesions on cherry immature fruitlets as well (M. Kałuźna, personal communication), leaving the role of this phytotoxin in pathogenicity open.

The positive trend between the reduced growth rate in rich medium and the pathogenicity led to the hypothesis that the inability of the identified *P. syringae* strains to cause disease was rather related to a metabolic impairment of those strains which does not allow them to reach population densities able to trigger disease [86]. The T3E screening revealed that all strains that resulted in a non-pathogenic phenotype on all three hosts were lacking a single T3E, namely HopAA, which has been shown to contribute to efficient formation of bacterial colonies *in planta* [92]. However, as *P. syringae* pv. phaseolicola strain 1448a is lacking this T3E as well, but was still growing at a higher growth rate and was pathogenic to cherry, peach and almond, this hypothesis can be rejected. Comparative genomics between closely related pathogenic and non-pathogenic strains revealed mutations within genes of metabolic pathways previously shown to be involved in virulence of plant pathogenic bacteria [93–97]. However, the role of those pathways in the pathogenicity of *Prunus* spp. is still unclear.

## Conclusion

Based on the obtained results, it is clear that the ability of *P. syringae* strains to cause disease on *Prunus* spp. is not the result of a common evolutionary event but is most probably due to an independent loss or gain of different factors in individual strains, not necessarily related to virulence. Moreover, the large number of strains found to be pathogenic on the tested hosts revealed that the *Prunus* spp. – *P. syringae* pathosystem does not represent the most suitable case for the investigation of virulence-related factors. A more comprehensive phenotyping and genome comparisons of both pathogen and host would provide more indications in order to reveal key factors in the pathogenicity of *P. syringae* on cherry, peach and almond.

## Additional file

Additional file 1: This file contains all supplementary tables and figures listed below. **Table S1.** List of phytotoxins and phytohormons screened in this study. **Table S1.** List of type III effectors (T3E) screened in this study and their respective locus tags in reference genomes. **Table S2.** Metrics of the PacBio RSII sequencing results. **Table S3.** Metrics of the Illumina MiSeq sequencing results for the two *Pseudomonas syringae* pv. persicae (Ppe) strains. **Table S4.** Metrics of the Illumina MiSeq sequencing results for the two *Pseudomonas* 11 *syringae* pv. persicae (Ppe) strains. **Figure S1.** Average nucleotide identity (ANI) matrix based on BLASTn and derived phylogeny of a set of the *Pseudomonas syringae* genomes used in this study. **Figure S2.** Pathogenicity test results performed on cherry immature fruitlets. (PDF 1680 kb)

## Abbreviations

ANIb: Average nucleotide identity based on BLASTN searches
CDS: Coding sequences
IAA: Indole acetic acid
MLSA: Multilocus sequence analysis
OD: Optical density
PG: Phylogroup
pv: Pathovar
T3E: Type III effectors
T3SS: Type III secretion system
