# Supplementary material for: Comparative genomics and pathogenicity potential of members of the Pseudomonas syringae species complex on Prunus spp
Source: BMC Genomics. 2019 Mar 5;20:172. doi: 10.1186/s12864-019-5555-y (PMC6402114; doi:10.1186/s12864-019-5555-y)
Supplement: Supplementary file 1 — This file contains all supplementary tables and figures listed below. Table S1. List of phytotoxins and phytohormons screened in this study. Table S1. List of type III effectors (T3E) screened in this study and their respective locus tags in reference genomes. Table S2. Metrics of the PacBio RSII sequencing results. Table S3. Metrics of the Illumina MiSeq sequencing results for the two Pseudomonas syringae pv. persicae (Ppe) strains. Table S4. Metrics of the Illumina MiSeq sequencing results for the two Pseudomonas 11 syringae pv. persicae (Ppe) strains. Figure S1. Average nucleotide identity (ANI) matrix based on BLASTn and derived phylogeny of a set of the Pseudomonas syringae genomes used in this study. Figure S2. Pathogenicity test results performed on cherry immature fruitlets. (PDF 1680 kb) [file 12864_2019_5555_MOESM1_ESM.pdf]

Supplemental tables and figures belonging to

Comparative genomics and pathogenicity potential of members of the *Pseudomonas syringae* species complex on *Prunus* spp.

Michela Ruinelli, Jochen Blom, Theo H.M. Smits and Joël F. Pothier

Table S1 List of phytotoxins and phytohormons screened in this study.

Table S2 List of type III effectors (T3E) screened in this study and their respective locus tags in reference genomes.

Table S3 Metrics of the PacBio RSII sequencing results.

Table S4 Metrics of the Illumina MiSeq sequencing results for the two *Pseudomonas syringae* pv. persicae (Ppe) strains.

Figure S1 Average nucleotide identity (ANI) matrix based on BLASTn and derived phylogeny of a set of the *Pseudomonas syringae* genomes used in this study.

Figure S2 Pathogenicity test results performed on cherry immature fruitlets.

Table S1 List of phytotoxins and phytohormons encoding genes screened in this study.

Product	Gene nomenclature	Reference Genome	NCBI locus tags	Reference
**Coronatine**	*corR*, *corS*, *corP*, *cmaA-E*, *cmaT*, *cmaU*, *cfa1-9*, *cfl*	AE016853.1	PSPTO_4680 to PSPTO_4690<br>and PSPTO_4704 to PSPTO_4712	[1-3]
**Cytokinin**	*ptz*	N.A.	CAA27315.1	[4]
**IAA conjugation**	*iaaL*	AE016853.1	PSPTO_0371	[1, 5, 6]
**IAA synthesis**	*iaaM*/*iaaH*	CP000075.1	Psyr_1536/Psyr_1537	[7, 8]
**Mangotoxin**	*mboA-F*	CP005970.1	PSYRMG_10110 to PSYRMG_10135	[9, 10]
**Phaseolotoxin**	N.D.	CP000058.1	PSPPH_4298 to PSPPH_4320	[11, 12]
**Syringolin**	*sylA-E*	CP000075.1	Psyr_1706 to Psyr_1702	[8, 13]
**Syringomycin**	*syrB1*, *syrB2*, *syrC*, *syrE*, *syrD*, *syrP*, *syrF*, *syrG*, *salA*	CP000075.1	Psyr_2613 to Psyr_2607<br>and Psyr_2602 to Psyr_2601	[8, 14-17]
**Syringopeptin**	*sypA-C*	CP000075.1	Psyr_2615 to Psyr_2614	[8, 18-20]
**Tabtoxin**	N.D.	AEAP01	PSYTB_28452 to PSYTB_28507	[21-23]<br>[24]
**Type III secretion system**	*hrp*/*hrc*	CP000058.1	PSPPH_1295 to PSPPH_1270	[25-27]
**Type III secretion system 2**	N.D.	CP000058.1	PSPPH_2515 to PSPPH _545	[28]

N.D.: not determined.

Table S2 List of type III effectors (T3E) screened in this study and their respective locus tags in reference genomes.

T3E	Locus tag on EDGAR	Reference genome	Reference
AvrA1	pv_aesculi_2250_3486	ACXT01	[29]
AvrB2	PSPPH_A0120	CP000058-CP000060	[27]
AvrB4	IYO_003600	CP011972-CP011973	[30]
AvrD1	IYO_003570	CP011972-CP011973	[30]
AvrE1	IYO_006770	CP011972-CP011973	[30]
AvrPto1	PSPTO_4001	AE016853.1-AE016855.1	[1]
AvrRpm1	IYO_008065	CP011972-CP011973	[30]
AvrRpm2	IYO_024217	CP011972-CP011973	[30]
AvrRps4	PSPPH_A0087	CP000058-CP000060	[27]
AvrRpt2	PTOT1_2469	ABSM01	[31]
HopA1	PSPTO_5354	AE016853.1-AE016855.1	[1]
HopAA1-1	PSPTO_1372	AE016853.1-AE016855.1	[1]
HopAA1-2	PSPTO_4718	AE016853.1-AE016855.1	[1]
HopAB1	PSPPH_A0127	CP000058-CP000060	[27]
HopAD1	PSPTO_4691	AE016853.1-AE016855.1	[1]
HopAE1	PSPPH_4326	CP000058-CP000060	[27]
HopAF1	PSPTO_1568	AE016853.1-AE016855.1	[1]
HopAG1	CFBP2118_04399	LT962481	this study
HopAH1	PSPTO_0905	AE016853.1-AE016855.1	[1]
HopAI1	PSPTO_0906	AE016853.1-AE016855.1	[1]
HopAJ1	PSPTO_0852	AE016853.1-AE016855.1	[1]
HopAK	PSPTO_4101	AE016853.1-AE016855.1	[1]
HopAL1	CFBP4215_01700	LT962480	this study
HopAM1-1	PSPTO_1022	AE016853.1-AE016855.1	[1]
HopAM1-2	PSPTO_A0005	AE016853.1-AE016855.1	[1]
HopAO1	PSPTO_4722	AE016853.1-AE016855.1	[1]
HopAO2	IYO_003720	CP011972-CP011973	[30]
HopAQ1	PSPTO_4703	AE016853.1-AE016855.1	[1]
HopAR1	CFBP2118_04979	LT962481	this study
HopAS1	PSPPH_4736	CP000058-CP000060	[27]
HopAT1	PSPPH_5225	CP000058-CP000060	[27]
HopAU1	PSPPH_A0031	CP000058-CP000060	[27]
HopAV1	PSPPH_A0056	CP000058-CP000060	[27]
HopAW1	IYO_003657	CP011972-CP011973	[30]
HopAY1	PSPPH_A0129	CP000058-CP000060	[27]
HopAZ1	IYO_018555	CP011972-CP011973	[30]
HopB1	PSPTO_1406	AE016853.1-AE016855.1	[1]
HopBB1-1	IYO_003727	CP011972-CP011973	[30]
HopBB1-2	IYO_003675	CP011972-CP011973	[30]
HopBC1	PSYAR_24412	AEAO01	[32]
HopBD1	PLA106_27566	AEAM01	[32]
HopBD2	NCPPB2254_03267	ODAM01000001-ODAM01000246	this study
HopBE1	PSYAR_10899	AEAO01	[32]
HopBF1	CFBP3840_p400101	LT963409-LT963413	this study

HopBG1	PMA4326_30242	AEAK01	[32]
HopBI1	PL963_p300075	LT963395-LT963401	this study
HopBJ1	N018_19725	CP007014-CP007015	N.A.
HopBK1	PSYRMG_01235	CP005970	[9]
HopBL1	CFBP3840_p100112	LT963409-LT963413	this study
HopBL2	CFBP3840_00659	LT963409-LT963413	this study
HopBM1	PSPTO_5633	AE016853.1-AE016855.1	[1]
HopBN1	IYO_013150	CP011972-CP011973	[30]
HopC1	PSPTO_0589	AE016853.1-AE016855.1	[1]
HopD1	PSPTO_0876	AE016853.1-AE016855.1	[1]
HopE1	PSPTO_4331	AE016853.1-AE016855.1	[1]
HopF3	PSPPH_3498	CP000058-CP000060	[27]
HopG1	PSPTO_4727	AE016853.1-AE016855.1	[1]
HopH1	PSPTO_0588	AE016853.1-AE016855.1	[1]
HopI1	IYO_005160	CP011972-CP011973	[30]
HopK1	PSPTO_0044	AE016853.1-AE016855.1	[1]
HopM1	PSPTO_1375	AE016853.1-AE016855.1	[1]
HopN1	IYO_006735	CP011972-CP011973	[30]
HopO1-1	PSPTO_A0018	AE016853.1-AE016855.1	[1]
HopO1-2	PSPTO_4594	AE016853.1-AE016855.1	[1]
HopP1	PSPTO_2678	AE016853.1-AE016855.1	[1]
HopQ1-1	PSPTO_0877	AE016853.1-AE016855.1	[1]
HopQ1-2	PSPTO_4732	AE016853.1-AE016855.1	[1]
HopR1	PSPTO_0883	AE016853.1-AE016855.1	[1]
HopS1	PLA106_22893	AEAM01	[32]
HopS2	IYO_004052	CP011972-CP011973	[30]
hopT1-1	PSPTO_A0019	AE016853.1-AE016855.1	[1]
hopT1-2	PSPTO_4593	AE016853.1-AE016855.1	[1]
HopU1	PSPTO_0501	AE016853.1-AE016855.1	[1]
HopV1	PSPTO_4720	AE016853.1-AE016855.1	[1]
hopX1	PSPTO_A0012	AE016853.1-AE016855.1	[1]
HopX2	A250_06861	CM002753-CM002754	[33]
HopY1	PSPTO_0061	AE016853.1-AE016855.1	[1]
HopZ3	IYO_029045	CP011972-CP011973	[30]
HopZ4	PLA107_32571	AEAF01	[32]
HopZ5	IYO_008282	CP011972-CP011973	[30]

Table S3 Metrics of the PacBio RSII sequencing results.

**Strain**[a]	**Pcerasi PL963**	**Pavii CFBP 3846**	**Pscer CFBP 6110**	**Pscer CFBP 6109**	**Pmp1 CFBP 3840**	**Pmp1 CFBP 2116**	**Pmp2 CFBP 3800**	**Pmp2 CFBP 6411**	**Psy CFBP 2118**	**Psy CFBP 4215**
**Number of SMRTcells**	6	6	5	6	5	6	6	5	5	6
**Total number of reads**	351,993	438,533	366,949	433,733	116,470	482,110	464,181	114,712	104,850	142,016
**Total number of bases**	2,040,283,420	2,364,983,321	2,903,342,251	1,846,300,53<br>4	899,391,466	2,500,388,640	2,748,099,409	635,868,141	706,366,439	802,154,567
**Mean read length (bp)**	5,796	5,392	7,912	4,256	7,636	5,186	5,920	5,543	6,736	5,648
**N_{50} read length (bp)**	9,563	9,716	11,239	7,583	11,001	8,825	9,738	8,429	10,106	8,373
**Number of mapped reads**	315,221	360,451	337,334	338,842	110,755	409,023	409,513	105,062	96,950	131,578
**Mean coverage (×)**	252.75	298.33	344.84	244.23	124.36	324.87	328.24	88.04	103.54	114.59
**Total number of contigs**	1Chr+6PP	1Chr+5PP	1Chr[b] +3PP	1Chr+3PP	1Chr[b]+4PP	1Chr+4PP	1Chr[b]+2PP	1Chr	1Chr	1Chr
**Size of Chr (bp)**	5,889,419	6,120,158	5,907,507	6,015,874	6,013,125	6,034,464	6,450,957	6,363,674	6,031,285	6,035,297
**G+C content (%) of Chr**	59.02	58.74	58.25	58.2	58.16	58.15	58.47	58.55	59.26	59.31
**CDS of Chr**	5,278	5,517	5,441	5,577	5,434	5,464	5,728	5,693	5,118	5,129
**Size of PP1 (bp)**	127,474	43,975[c,d]	127,142[c]	118,774[c,d]	110,420[d]	19,743[c]	83,341[d]			
**G+C content (%) of PP1**	54.82	56.04	55.32	55.48	54.65	53.35	56.28			
**CDS PP1**	140	57	143	149	137	30	87			
**Size of PP2 (bp)**	144,075[d]	109,843[d]	94,021[d]	86,328[d]	90,464	61,361[c,d]	67,760[c,d]			
**G+C content (%) of PP2**	55.58	55.74	55.96	56.26	55.04	55.18	55.31			
**CDS PP2**	155	125	110	95	115	67	96			
**Size of PP3 (bp)**	81,323[d]	108,842[d]	103,529[d]	111,777[d]	86,369[d]	83,816				
**G+C content (%) of PP3**	55.11	55.79	56.51	56.6	58.26	54.81				
**CDS PP3**	94	128	108	114	84	104				
**Size of PP4 (bp)**	70,589[d]	77,492[d]			78,348	82,845[d]				
**G+C content (%) of PP4**	55.2	55.94			55.2	55.87				
**CDS PP4**	81	100			104	103				
**Size of PP5 (bp)**	48,121[d]	41,285[d]								
**G+C content (%) of PP5**	55.02	54.5								
**CDS PP5**	67	68								
**Size of PP6 (bp)**	18,360[d]									
**G+C content (%) of PP6**	55.34									
**CDS PP6**	29									
**Total genome size (bp)**	6,379,361	6,501,595	6,232,199	6,332,753	6,378,726	6,282,229	6,602,058	6,363,674	6,031,285	6,035,297
**Total number of CDS**	5,844	5,995	5,802	5,935	5,874	5,768	5,911	5,693	5,118	5,129
**NCBI accession numbers**	LT963395-<br>LT963401	LT963402-<br>LT963407	OLMP01000001-<br>OLMP01000002<br>LT985210-<br>LT985212	LT963391-<br>LT963394	LT963409-<br>LT963413	LT985192-LT985195<br>OLMD01000001-<br>OLMD01000002	OLMQ01000001-<br>OLMQ01000003<br>LT985190-<br>LT98519	LT963408	LT962481	LT962480

[a] Strains abbreviation code: see Table 2.1; [b] Chromosome (Chr) is circularized based on other genomes; [c] Putative plasmid (PP) is not circularized; [d] PP possessed a plasmid replication protein encoding gene *repA*.

Table S4 Metrics of the Illumina MiSeq sequencing results for the two *Pseudomonas syringae* pv. persicae (Ppe) strains.

	Ppe CFBP 1573	Ppe NCPPB 2254
**Total number of reads**	1,948,134	935,008
**Total number of contigs**	214	246
**Max. contig length (bp)**	206,399	210,561
**Min. contig length (bp)**	549	536
**Mean contig length (bp)**	30,187	26,114
**N_{50} read length(bp)**	67,876	64,693
**Mean coverage (×)**	61	43
**Genome size (bp)**	6,460,112	6,423,988
**G+C content (%)**	58.5	58.8
**# CDS**	6,079	5,990
**NCBI accession numbers**	ODAL01000001-ODAL01000214	ODAM01000001-ODAM01000246

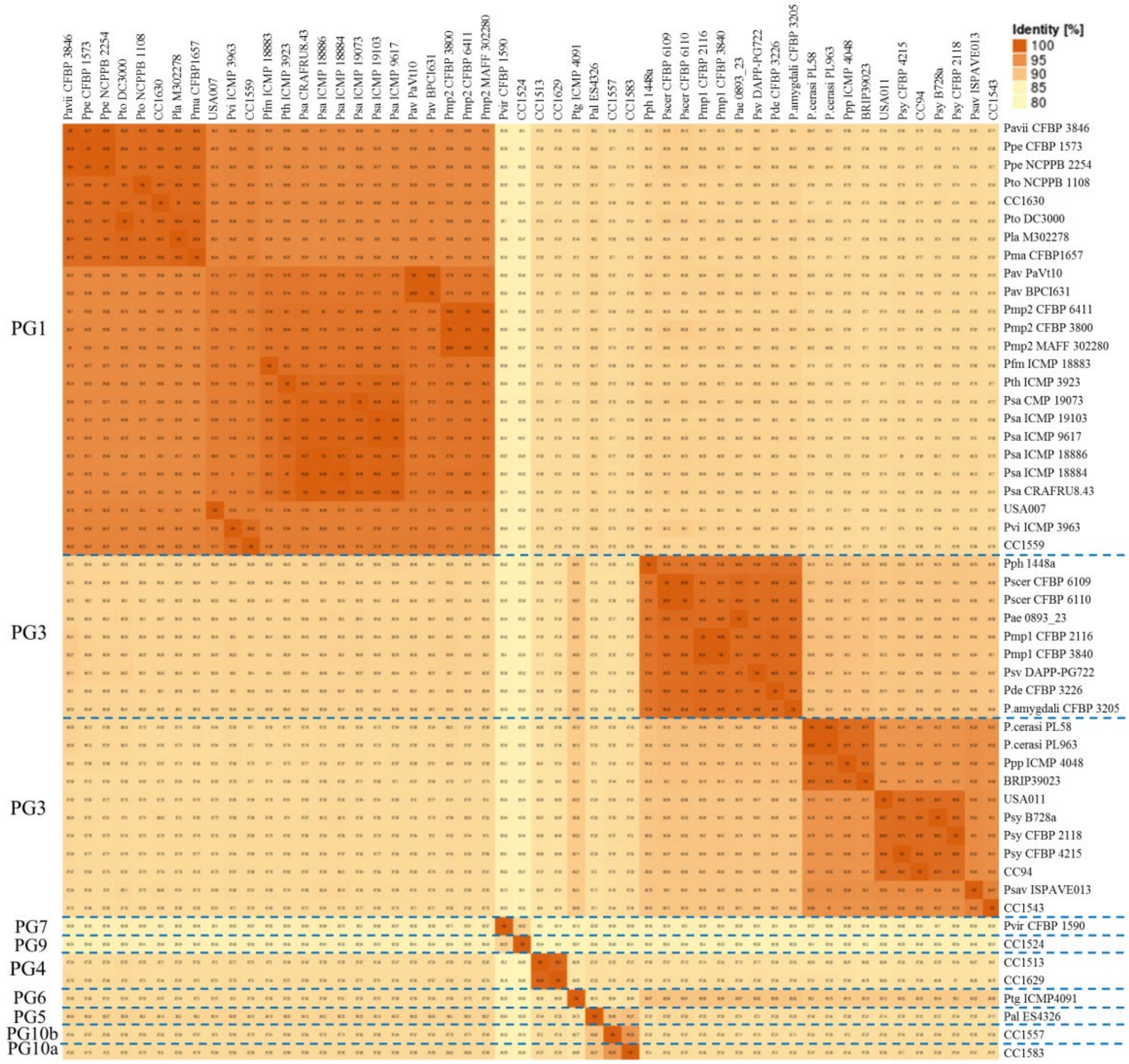

Figure S1 Average nucleotide identity (ANI) matrix based on BLASTn and derived phylogeny of a set of the *Pseudomonas syringae* genomes used in this study. Phylogroups (PG) are indicated on the left, strain names correspond to the code field from Table 1. Horizontal dashed lines represent groups of strains belonging to the same species based on the ANI species threshold of 95%. ANI matrix and figure were generated using EDGAR v.2.2 [34].

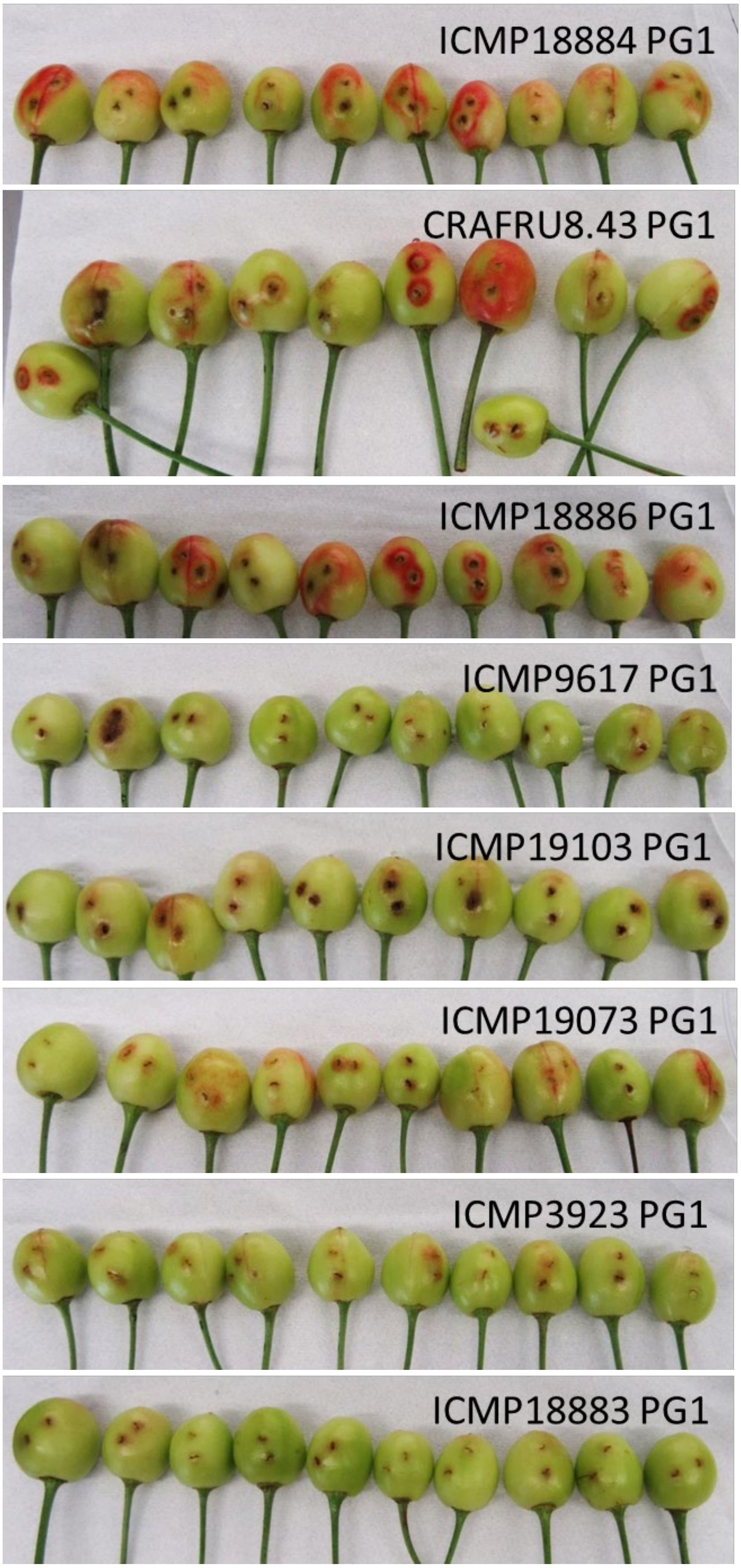
ICMP18884 PG1
CRAFRU8.43 PG1
ICMP18886 PG1
ICMP9617 PG1
ICMP19103 PG1
ICMP19073 PG1
ICMP3923 PG1
ICMP18883 PG1

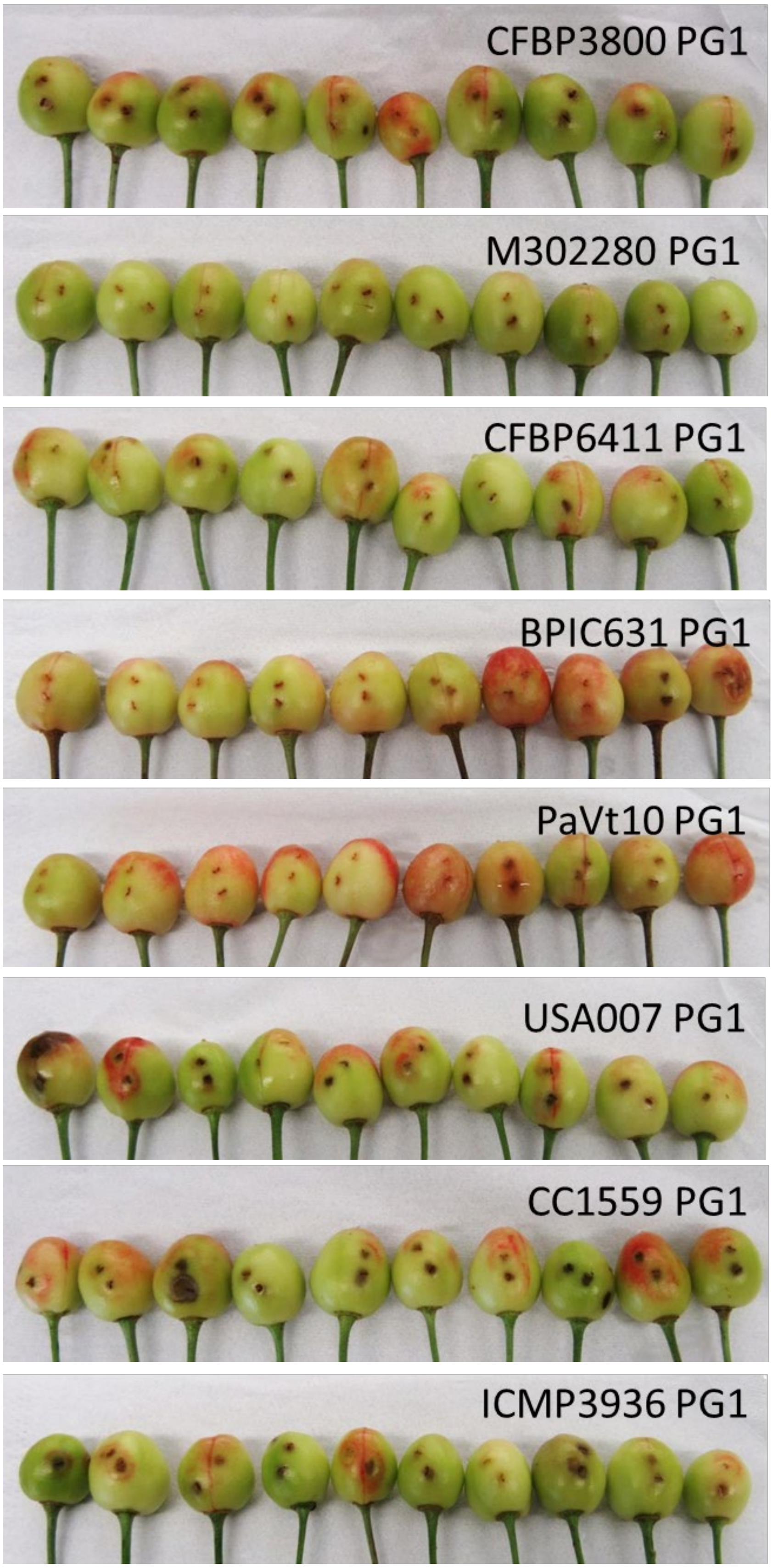
CFBP3800 PG1
M302280 PG1
CFBP6411 PG1
BPIC631 PG1
PaVt10 PG1
USA007 PG1
CC1559 PG1
ICMP3936 PG1

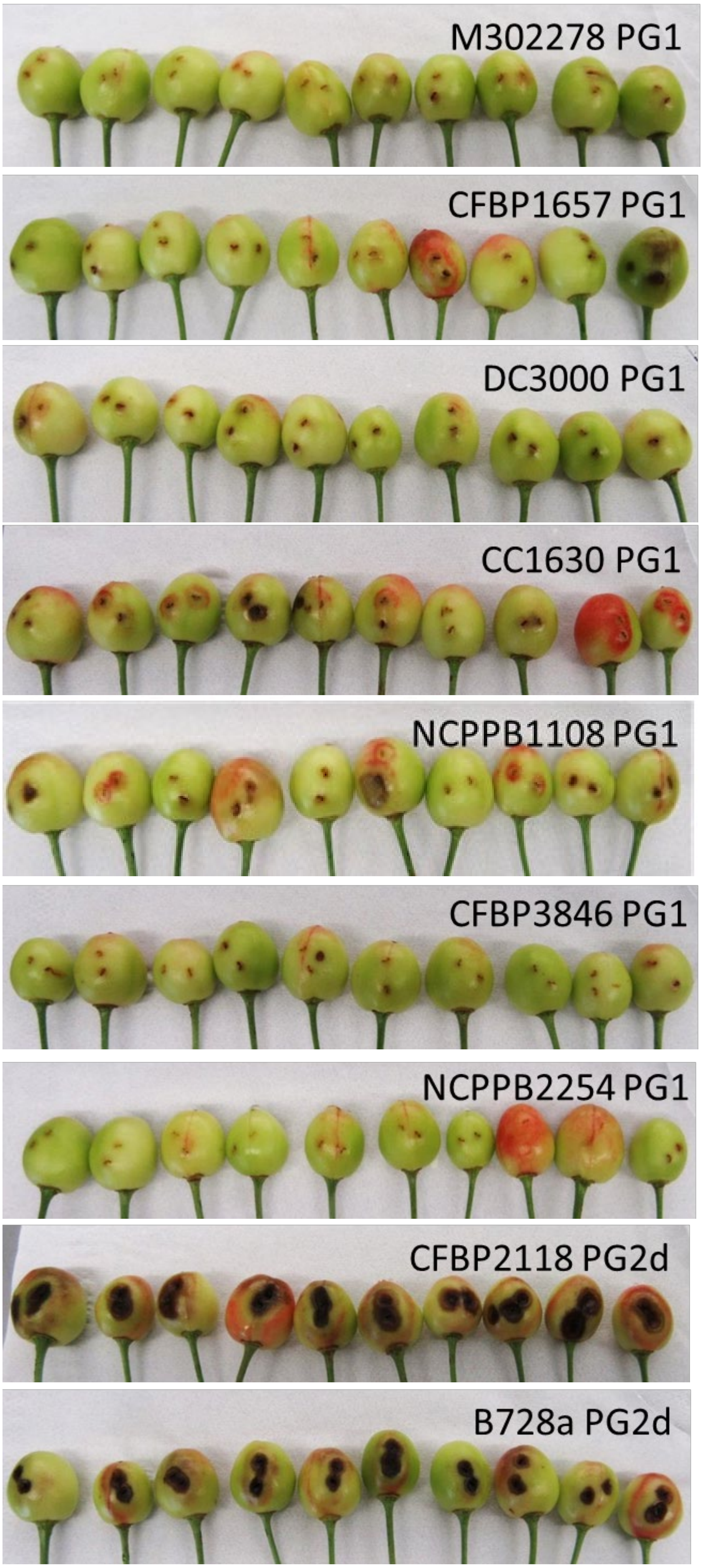
M302278 PG1
CFBP1657 PG1
DC3000 PG1
CC1630 PG1
NCPPB1108 PG1
CFBP3846 PG1
NCPPB2254 PG1
CFBP2118 PG2d
B728a PG2d

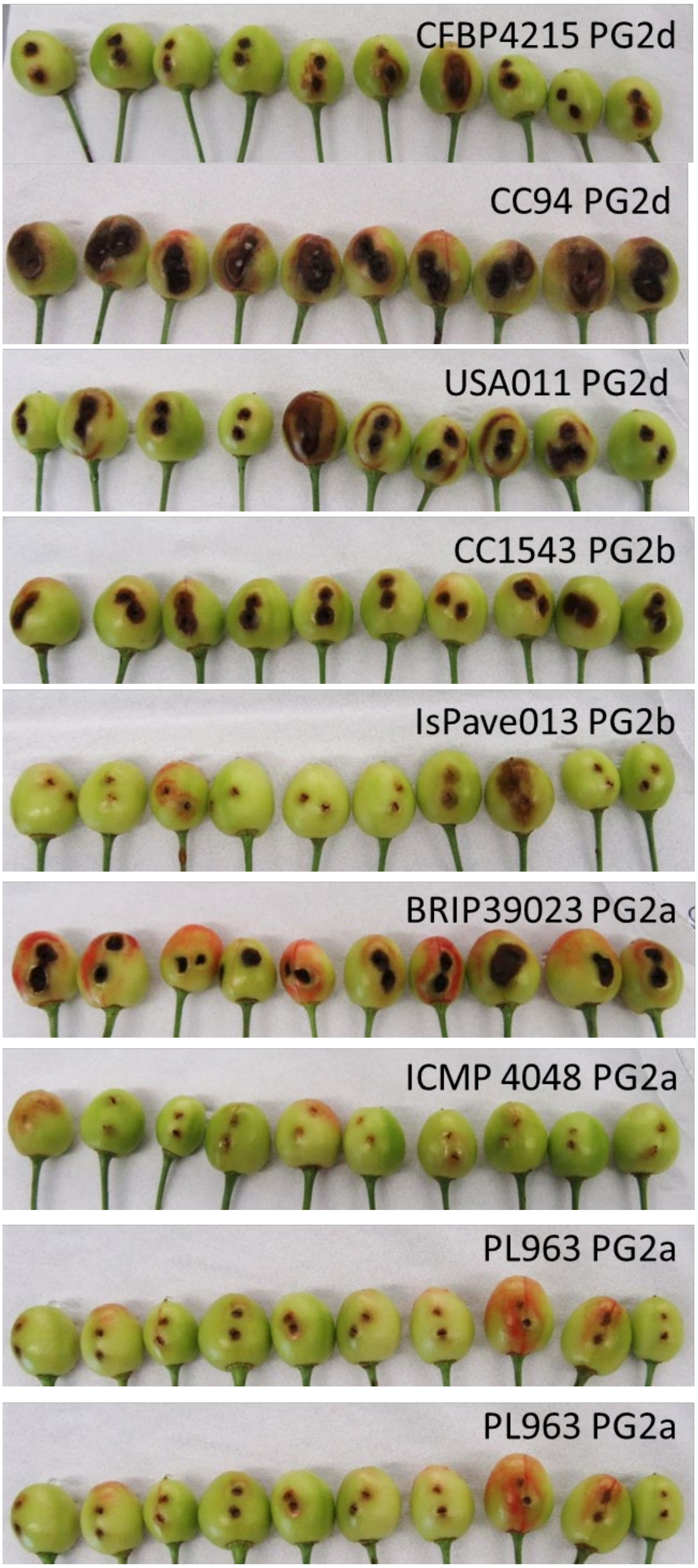
CFBP4215 PG2d
CC94 PG2d
USA011 PG2d
CC1543 PG2b
IsPave013 PG2b
BRIP39023 PG2a
ICMP 4048 PG2a
PL963 PG2a
PL963 PG2a

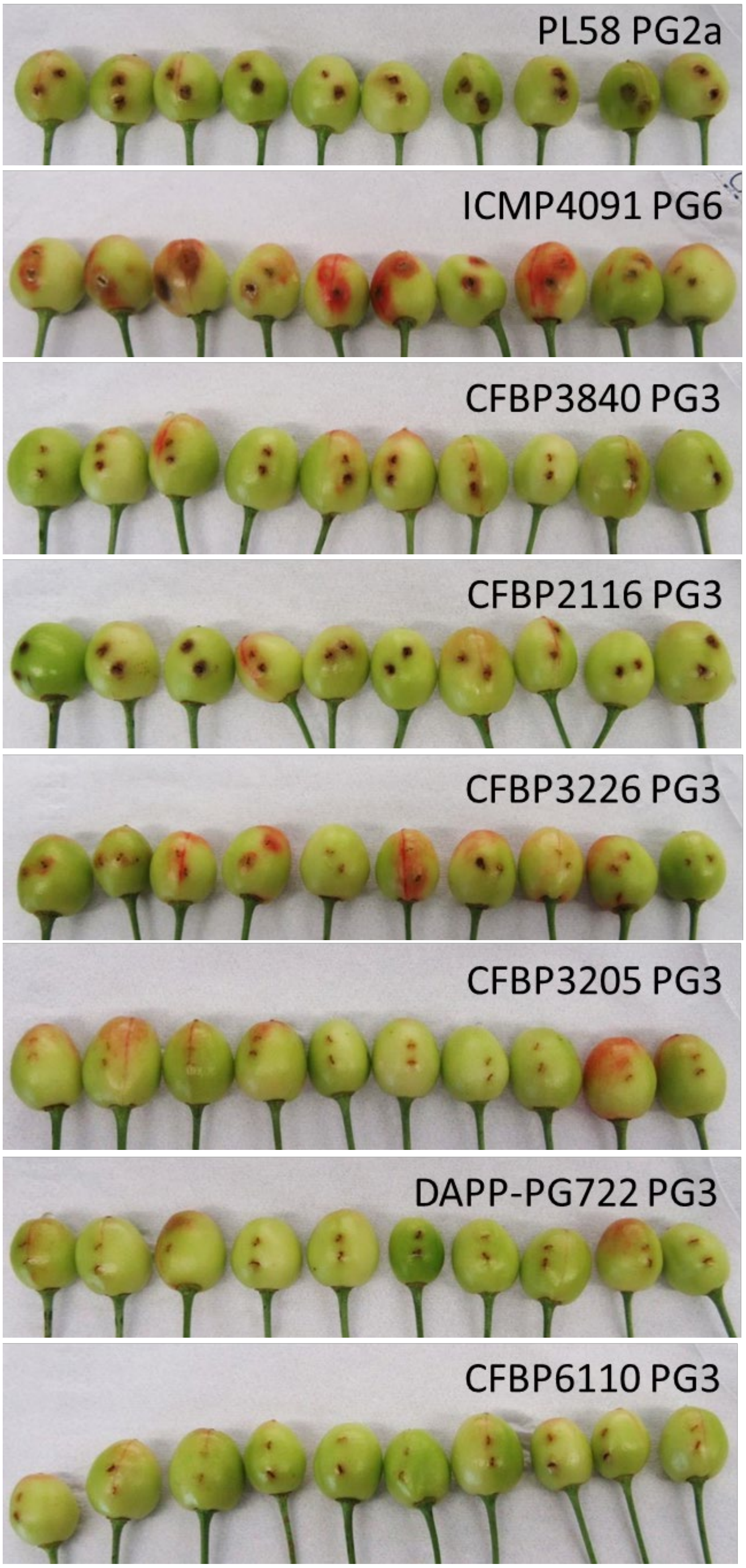
PL58 PG2a
ICMP4091 PG6
CFBP3840 PG3
CFBP2116 PG3
CFBP3226 PG3
CFBP3205 PG3
DAPP-PG722 PG3
CFBP6110 PG3

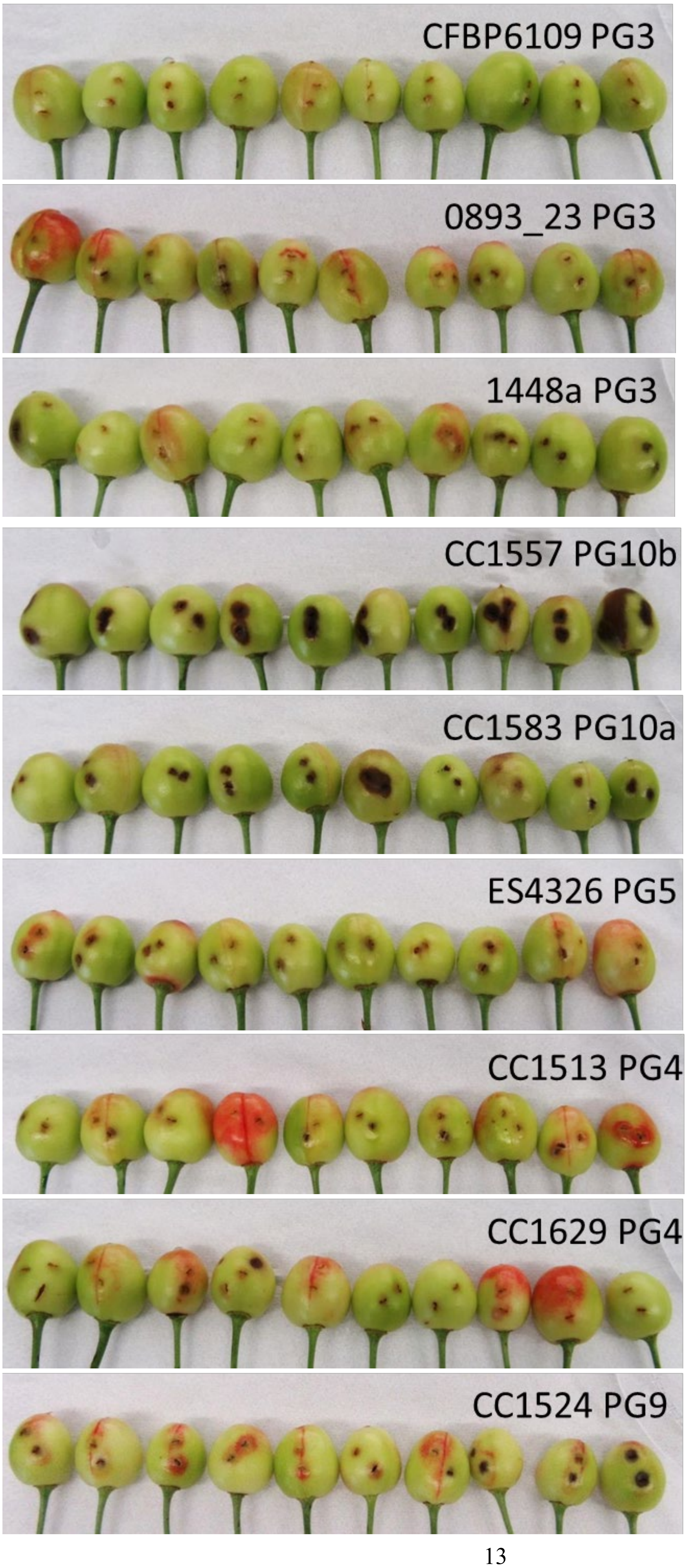
CFBP6109 PG3
0893_23 PG3
1448a PG3
CC1557 PG10b
CC1583 PG10a
ES4326 PG5
CC1513 PG4
CC1629 PG4
CC1524 PG9

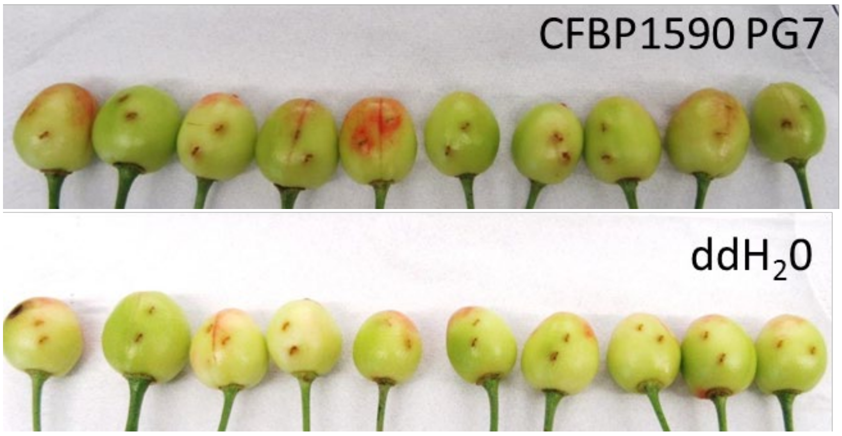

Figure S2 Pathogenicity test results performed on cherry immature fruitlets.

References

1. Buell CR, Joardar V, Lindeberg M, Selengut J, Paulsen IT, Gwinn ML, Dodson RJ, Deboy RT, Durkin AS, Kolonay JF *et al*: The complete genome sequence of the *Arabidopsis* and tomato pathogen *Pseudomonas syringae* pv. tomato DC3000. Proc Natl Acad Sci USA. 2003; 100: 10181-6.
2. Zheng X-y, Spivey Natalie W, Zeng W, Liu P-P, Fu Zheng Q, Klessig Daniel F, He Sheng Y, Dong X: Coronatine promotes *Pseudomonas syringae* virulence in plants by activating a signaling cascade that inhibits salicylic acid accumulation. Cell Host Microbe. 2012; 11: 587-96.
3. Lee S, Ishiga Y, Clermont K, Mysore Kirankumar S: Coronatine inhibits stomatal closure and delays hypersensitive response cell death induced by nonhost bacterial pathogens. PeerJ. 2013; 1: e34.
4. Powell GK, Morris RO: Nucleotide sequence and expression of a *Pseudomonas savastanoi* cytokinin biosynthetic gene: homotogy with *Agrobacterium tumefaciens tmr* and *tzs* loci. Nucleic Acids Res. 1986; 14: 2555-65.
5. Glass NL, Kosuge T: Cloning of the gene for indoleacetic acid-lysine synthetase from *Pseudomonas syringae* subsp. savastanoi. J Bacteriol. 1986; 166: 598-603.
6. Glass NL, Kosuge T: Role of indoleacetic acid-lysine synthetase in regulation of indoleacetic acid pool size and virulence of *Pseudomonas syringae* subsp. savastanoi. J Bacteriol. 1988; 170: 2367-73.
7. Kosuge T, Heskett MG, Wilson EE: Microbial synthesis and degradation of indole-3-acetic acid. I. The conversion of L-tryptophan to indole-3-acetamide by an enzyme system from *Pseudomonas savastanoi*. J Biol Chem. 1966; 241: 3738-44.
8. Feil H, Feil WS, Chain P, Larimer F, DiBartolo G, Copeland A, Lykidis A, Trong S, Nolan M, Goltsman E *et al*: Comparison of the complete genome sequences of *Pseudomonas syringae* pv. syringae B728a and pv. tomato DC3000. Proc Natl Acad Sci USA. 2005; 102: 11064-9.
9. Martínez-García PM, Rodríguez-Palenzuela P, Arrebola E, Carrión VJ, Gutiérrez-Barranquero JA, Pérez-García A, Ramos C, Cazorla FM, de Vicente A: Bioinformatics analysis of the complete genome sequence of the mango tree pathogen *Pseudomonas syringae* pv. syringae UMAF0158 reveals traits relevant to virulence and epiphytic lifestyle. PLoS One. 2015; 10: e0136101.
10. Arrebola E, Cazorla FM, Durán VE, Rivera E, Olea F, Codina JC, Pérez-García A, de Vicente A: Mangotoxin: a novel antimetabolite toxin produced by *Pseudomonas syringae* inhibiting ornithine/arginine biosynthesis. Physiol Mol Plant Pathol. 2003; 63: 117-27.
11. Arai T, Kino K: A novel L-amino acid ligase is encoded by a gene in the phaseolotoxin biosynthetic gene cluster from *Pseudomonas syringae* pv. phaseolicola 1448A. Biosci Biotechnol Biochem. 2008; 72: 3048-50.
12. Patil SS, Tam LQ: Mode of action of the toxin from *Pseudomonas phaseolicola*: I. Toxin specificity, chlorosis, and ornithine accumulation. Plant Physiol. 1972; 49: 803-7.
13. Schellenberg B, Ramel C, Dudler R: *Pseudomonas syringae* virulence factor syringolin A counteracts stomatal immunity by proteasome inhibition. Mol Plant-Microbe Interact. 2010; 23: 1287-93.
14. Hutchison ML, Gross DC: Lipopeptide phytotoxins produced by *Pseudomonas syringae* pv. syringae: Comparison of the biosurfactant and ion channel-forming activities of syringopeptin and syringomycin. Mol Plant-Microbe Interact. 1997; 10: 347-54.
15. Mott KA, Takemoto JY: Syringomycin, a bacterial phytotoxin, closes stomata. Plant Physiol. 1989; 90: 1435-9.
16. Bender CL, Alarcón-Chaidez F, Gross DC: *Pseudomonas syringae* phytotoxins: Mode of action, regulation, and biosynthesis by peptide and polyketide synthetases. Microbiol Mol Biol Rev. 1999; 63: 266-92.
17. Vaughn VL, Gross DC: Characterization of *salA*, *syrF* and *syrG* genes and attendant regulatory networks involved in plant pathogenesis by *Pseudomonas syringae* pv. syringae B728a. PLoS One. 2016; 11: e0150234.
18. Di Giorgio D, Camoni L, Ballio A: Toxins of *Pseudomonas syringae* pv. syringae affect H+-transport across the plasma membrane of maize. Physiol Plant. 1994; 91: 741-6.

19. Di Giorgio D, Camoni L, Mott KA, Takemoto JY, Ballio A: Syringopeptins, *Pseudomonas syringae* pv. syringae phytotoxins, resemble syringomycin in closing stomata. Plant Pathol. 1996; 45: 564-71.
20. Iacobellis NS, Lavermicocca P, Grgurina I, Simmaco M, Ballio A: Phytotoxic properties of *Pseudomonas syringae* pv. syringae toxins. Physiol Mol Plant Pathol. 1992; 40: 107-16.
21. Stewart WW: Isolation and proof of structure of wildfire toxin. Nature. 1971; 229: 174-8.
22. Turner JG: Tabtoxin, produced by *Pseudomonas tabaci,* decreases *Nicotiana tabacum* glutamine synthetase *in vivo* and causes accumulation of ammonia. Physiol Plant Pathol. 1981; 19: 57-67.
23. Thomas MD, Langston-Unkefer PJ, Uchytil TF, Durbin RD: Inhibition of glutamine synthetase from pea by tabtoxinine-beta-lactam. Plant Physiol. 1983; 71: 912-5.
24. Kinscherf TG, Willis DK: The biosynthetic gene cluster for the beta-lactam antibiotic tabtoxin in *Pseudomonas syringae*. J Antibiot. 2005; 58: 817-21.
25. Lindgren PB, Peet RC, Panopoulos NJ: Gene cluster of *Pseudomonas syringae* pv. "phaseolicola" controls pathogenicity of bean plants and hypersensitivity of nonhost plants. J Bacteriol. 1986; 168: 512-22.
26. Lindgren PB, Panopoulos NJ, Staskawicz BJ, Dahlbeck D: Genes required for pathogenicity and hypersensitivity are conserved and interchangeable among pathovars of *Pseudomonas syringae*. Mol Gen Genet. 1988; 211: 499-506.
27. Joardar V, Lindeberg M, Jackson RW, Selengut J, Dodson R, Brinkac LM, Daugherty SC, Deboy R, Durkin AS, Giglio MG *et al*: Whole-genome sequence analysis of *Pseudomonas syringae* pv. phaseolicola 1448A reveals divergence among pathovars in genes involved in virulence and transposition. J Bacteriol. 2005; 187: 6488-98.
28. Gazi AD, Sarris PF, Fadouloglou VE, Charova SN, Mathioudakis N, Panopoulos NJ, Kokkinidis M: Phylogenetic analysis of a gene cluster encoding an additional, rhizobial-like type III secretion system that is narrowly distributed among *Pseudomonas syringae* strains. BMC Microbiol. 2012; 12: 188.
29. Green S, Studholme DJ, Laue BE, Dorati F, Lovell H, Arnold D, Cottrell JE, Bridgett S, Blaxter M, Huitema E *et al*: Comparative genome analysis provides insights into the evolution and adaptation of *Pseudomonas syringae* pv. aesculi on *Aesculus hippocastanum*. PLoS One. 2010; 5: e10224.
30. Templeton MD, Warren BA, Andersen MT, Rikkerink EH, Fineran PC: Complete DNA sequence of *Pseudomonas syringae* pv. actinidiae, the causal agent of kiwifruit canker disease. Genome Announc. 2015; 3: e01054-15.
31. Almeida NF, Yan S, Lindeberg M, Studholme DJ, Schneider DJ, Condon B, Liu H, Viana CJ, Warren A, Evans C: A draft genome sequence of *Pseudomonas syringae* pv. *tomato* T1 reveals a type III effector repertoire significantly divergent from that of *Pseudomonas syringae* pv. tomato DC3000. Mol Plant-Microbe Interact. 2009; 22.
32. Baltrus DA, Nishimura MT, Romanchuk A, Chang JH, Mukhtar MS, Cherkis K, Roach J, Grant SR, Jones CD, Dangl JL: Dynamic evolution of pathogenicity revealed by sequencing and comparative genomics of 19 *Pseudomonas syringae* isolates. PLoS Pathog. 2011; 7: e1002132.
33. McCann HC, Rikkerink EHA, Bertels F, Fiers M, Lu A, Rees-George J, Andersen MT, Gleave AP, Haubold B, Wohlers MW *et al*: Genomic analysis of the kiwifruit pathogen *Pseudomonas syringae* pv. actinidiae provides insight into the origins of an emergent plant disease. PLoS Pathog. 2013; 9: e1003503.
34. Blom J, Kreis J, Spänig S, Juhre T, Bertelli C, Ernst C, Goesmann A: EDGAR 2.0: an enhanced software platform for comparative gene content analyses. Nucleic Acids Res. 2016; 44: W22-8.
